# Changes in incidence and epidemiology of antimicrobial resistant pathogens before and during the COVID-19 pandemic in Germany, 2015–2022

**DOI:** 10.1186/s12866-024-03723-5

**Published:** 2025-01-28

**Authors:** Jonathan H. J. Baum, Achim Dörre, Felix Reichert, Ines Noll, Marcel Feig, Tim Eckmanns, Mirco Sandfort, Sebastian Haller

**Affiliations:** 1 Department of Infectious Disease Epidemiology, Robert Koch Institute (RKI), Berlin, Germany; 2 Postgraduate Training for Applied Epidemiology (PAE), Department of Infectious Disease Epidemiology, Robert Koch Institute (RKI), Berlin, Germany; 3 ECDC Fellowship Programme, Field Epidemiology path (EPIET), European Centre for Disease Prevention and Control (ECDC), Stockholm, Sweden; 4 Department of Method Development, Research Infrastructure and Information Technology, Robert Koch Institute (RKI), Berlin, Germany

**Keywords:** Drug resistance, Antimicrobial resistance, Acinetobacter, Klebsiella pneumoniae, Escherichia coli, Methicillin-resistant staphylococcus aureus, COVID-19

## Abstract

**Background:**

Carbapenem-resistant Gram-negative bacteria and methicillin-resistant *Staphylococcus aureus* (MRSA) are among WHO’s priority pathogens with antimicrobial resistance (AMR). Studies suggest potential impacts of the COVID-19-pandemic on AMR. We described changes in AMR incidence and epidemiology in Germany during the COVID-19-pandemic.

**Methods:**

We used two independent datasets, statutory surveillance and laboratory-based Antibiotic Resistance Surveillance (ARS). We included statutory notifications of infections/colonisations of carbapenem-resistant *Acinetobacter* spp., *Klebsiella pneumoniae*, *Escherichia coli* (CRA/CRKP/CREC) and invasive MRSA. Using Poisson/negative binomial regression and assuming continued pre-pandemic (2015/2017–2020) trends, we projected hypothetical notifications as if the pandemic had not occurred. We quantified annual changes during the pandemic period (2020–2022) by comparing to observed notifications. Additional models considered inpatient reductions, seasonality, infections only, or resistant isolates from ARS.

**Results:**

CRA notified cases were reduced by -30% (95%CI -39%|-20%) in 2020, -23% (-36%|-8%) in 2021, but + 32% (+ 6%|+64%) higher in 2022 relative to hypothetical pre-pandemic projections. Changes were − 35%/-31%/+6% for CRKP, -40%/-61%/-48% for CREC and − 33%/-25%/-20% for MRSA. Statutory-models accounting for fewer inpatients, seasonality and infections only showed similar trends, as did ARS-models for resistant isolates and infections. International mobility for CRA, CRKP and CREC decreased in 2020–2021, then increased in 2022.

**Conclusions:**

We observed significant reductions of AMR notifications and infections during 2020–2021, also when accounting for fewer inpatients. We conclude a genuine reduction of AMR spread occurred during the pandemic. Factors like fewer hospitalisations and reduced international mobility contributed. Rising international mobility may partly explain increases for CRA, CRKP and CREC in 2022. A solid understanding of AMR trends improves infection prevention and control.

**Supplementary Information:**

The online version contains supplementary material available at 10.1186/s12866-024-03723-5.

## Introduction

Antimicrobial resistance (AMR) is one of the biggest global health threats according to the World Health Organisation [1]. Global studies for 2019 estimate nearly 4.95 million deaths associated with, and at least 1.27 million deaths directly attributable to AMR [2]. In Germany, 45,700 deaths were associated with, and 9,650 deaths attributable to AMR in 2019 [2, 3]. Resistance, particularly against last-line antibiotics such as carbapenems remains a challenge, limits treatment options and contributes to fatal outcomes [4]. In addition to the individual and public health burden, AMR has a considerable economic impact [5].

In Germany, the mandatory national surveillance covers carbapenem-resistant *Acinetobacter* spp. (CRA), invasive methicillin-resistant *Staphylococcus aureus* (MRSA) and carbapenem-resistant Enterobacterales [6]. The surveillance and the taxonomic order of Enterobacterales comprise diverse genera, including the most common, carbapenem-resistant *Klebsiella pneumoniae* (CRKP) and *Escherichia coli* (CREC) [7, 8]. In the antimicrobial resistance surveillance report of the European Centre for Disease Prevention and Control (ECDC) the species *Escherichia coli*, *Klebsiella pneumoniae* and *Staphylococcus aureus* reported most frequently among EU/EEA countries participating in the EARS-Net surveillance [7]. These were also among the most frequently reported species in the European point prevalence survey (PPS) on healthcare associated infections 2016/17, as well as in the most recent PPS 2022/23 [8, 9].

Since the early 2020 detection of Severe Acute Respiratory Syndrome Corona Virus 2 (SARS-CoV-2) [10], the Coronavirus Disease 2019 (COVID-19) pandemic affected all aspects of society, particularly the healthcare system. Potential impacts of the COVID-19 pandemic and subsequent interventions on AMR have been discussed [11–14]. Overwhelmed health systems, (over)prescription of antibiotics during COVID-19 treatment, and disruptions in infection prevention and control programs are examples of potentially contributing factors for AMR spread. Contrarily, non-pharmaceutical interventions like increased hygiene practices and social distancing may have reduced AMR pathogen incidence. Reduced non-COVID-19 in- and outpatient numbers, as well as fewer elective surgeries and patient transfers, could have contributed to slowing AMR spread. Initial analyses for Germany indicated a decrease in CRA, MRSA and Enterobacterales notifications in 2020 [15].

Enhancing our understanding of COVID-19’s impact on AMR epidemiology in Germany is crucial for informing AMR management. Therefore, we described the epidemiology of CRA, CRKP, CREC and MRSA in Germany before and during the COVID-19 pandemic. We focused on pathogens in the “critical” or “high” group of the 2024 *WHO Bacterial Priority Pathogens List* [16]. We quantified changes in annual incidences during the pandemic by comparing observed incidences with hypothetical projections, based on continued pre-pandemic trends and assuming no pandemic.

## Methods

### Study type and study period

We performed retrospective descriptive analyses of AMR pathogen incidences before and during the COVID-19 pandemic, analysing CRA, MRSA, CRKP and CREC. Starting with the introduction of the notification requirements, our study period included complete weeks between 2 January 2017 (only MRSA: 5 January 2015) and 25 December 2022. We specified pre-pandemic (01/2015–12/2019) and pandemic (01/2020–12/2022) periods. Although Germany initially reported cases in February 2020 [10], we included the whole year as pandemic period to obtain comparable annual intervals.

### Primary study database, study population and case definitions

Our main database was the German statutory notification surveillance system, which records mandatory CRA, CRKP, CREC and invasive MRSA notifications by laboratories according to § 7 Infection Protection Act (IfSG) [6]. The study population included cases fulfilling § 11 [2] IfSG [6, 17] case definitions, with notification date in the study period. Cases were defined as individuals with newly detected CRA, CRKP and CREC infections or colonisations with a carbapenemase or reduced phenotypic sensitivity. MRSA cases were defined as new *Staphylococcus aureus* infections in blood or cerebrospinal fluid with mecA-gene detection or phenotypic methicillin resistance.

We included COVID-19 cases in Germany according to the statutory case definition [17], along with population data and annual hospital inpatient counts from the German Federal Statistical Office (DESTATIS) [18].

### Secondary study database, study population, and case definitions

We used the Antibiotic Resistance Surveillance (ARS) as our secondary database [19]. ARS is a laboratory-based surveillance network comprising data of about one third of German hospitals. Results of their routine antibiotic susceptibility testing are voluntarily transferred by respective laboratories to the Robert Koch Institute.

Analogous to statutory case definitions, we considered isolates resistant upon classification as resistant (“R”) or susceptible upon increased exposure (“I”) to imipenem or meropenem for CRA, CRKP and CREC and resistant (“R”) to oxacillin or flucloxacillin for MRSA. Between 2017 and 2022 we included the first isolate per patient per quarter (copy-strain-rule) from laboratories that participated in ARS throughout 2017–2022. We excluded screening isolates.

### Data and descriptive epidemiology

The following information was available for notifications: pathogen, notification date, age, federal state, infection/colonisation, mobility history, hospitalization date(s), sample date(s), sample material(s), death, if cases belonged to an outbreak, and outbreak setting.

We considered cases with pathogen detection in blood, cerebrospinal fluid, bronchoalveolar lavage, urine or wound samples as infected, and cases with detection in stool or screening samples as colonised. We defined the presumed acquisition setting based on a combination of statutory variables: infection/colonisation, mobility history, hospitalisation date(s), sample date(s), sample material(s), outbreak involvement and outbreak setting. We distinguished “probably nosocomial”, “possibly nosocomial”, “possibly community”, “possibly international” and “missing”.

We categorised mobility history into “domestic”, “international“ (i.e. at least one international stay possibly relevant for exposure), and “missing”. To analyse international mobility, we selected the top five reported countries per CRA, CRKP and CREC. For CRKP less frequent countries were grouped as “other”. We excluded MRSA from this sub-analysis due to the limited number of documented prior international stays.

We described notified cases by monthly incidence, sex and age groups, federal states, infection/colonisation, acquisition setting, outcome death (for infected cases only) and (international) mobility in pre-pandemic and pandemic periods. Due to varying case definitions, we excluded MRSA from sub-analyses regarding infection/colonisation and acquisition setting. Moreover, we plotted weekly pathogen counts and SARS-CoV-2 notifications together.

For ARS isolates, we analysed pathogen, date, copy-strain-rule, care type, sample material and results of resistance testing for imipenem and meropenem (CRA, CREC, CRKP) or oxacillin and flucloxacillin (MRSA). We identified isolates from infections based on the sample material types blood culture, respiratory, punctate, urine or wound.

### Statistical analyses and modelling

To quantify changes in incidence before and during the pandemic, we (a) fitted regression models that reflected observed annual incidences throughout the study period (2015/2017–2022), (b) used these to project annual incidence in the pandemic period (2020–2022) while assuming pre-pandemic trends continued, and (c) compared observed with projected incidence rate ratios (IRR) per pandemic year. We interpreted them as relative changes, i.e. as relative reductions (negative values) or increases (positive values) of observed incidence relative to hypothetical projections. We modelled the hypothetical annual incidences in the pandemic years by: (a) building the regression models based on the entire study period with a dummy covariate that was 0 for pre-pandemic and 1, 2 or 3 for pandemic years and (b) re-running the regression models with the dummy covariate set to 0 for all years (Supplementary Text 1). 95% confidence intervals (95% CI) and p-values (p) below 5% indicated statistical significance.

We employed 4 modelling approaches: In a primary annual model, we fitted Poisson or negative binomial regression models to annual case numbers and assessed them based on likelihood-ratio-tests. A second annual model additionally accounted for the concurrent reduction in hospitalization by including hospital inpatient numbers as an offset. To reflect pathogen seasonality and sub-annual trends, a third weekly model in an interrupted time series analysis design was fitted to weekly notifications. We used Poisson regression for baseline trend and sine/cosine terms for seasonality, informed by periodograms of weekly notifications. As before, an additional fourth weekly model accounted for the concurrent hospital inpatient count (Supplementary Text 1).

With ARS data, we employed the primary annual models for all isolates, resistant isolates, resistant isolates from infections only and resistant isolates stratified by inpatient or outpatient care type. We also analysed the percentage of resistance, relative to all isolates.

We compared sociodemographics and assessed changes in the distribution of categorical variables between the pre-pandemic and the pandemic period 2020–2021 using the chi-squared statistical test. For this analysis we excluded 2022 from the pandemic period due to relatively different AMR trends.

### Statistical software

We used R (4.2.2) [20] and RStudio (2023.03.0.B.386) [21], including packages *tidyverse*, *lubridate*, *ISOweek*, *tsibble*, *MASS*, *trending*, *lmtest*, *TSA*, *slider*, *ggalluvial*, *ggh4x*, *RColorBrewer*, *gtsummary* and *flextable*.

## Results

### Changes in sociodemographic characteristics and international mobility

We included *n* = 4,079 CRA, *n* = 9,347 CRKP, *n* = 4,686 CREC and *n* = 17,090 MRSA statutory notifications. Table 1 summarises notification characteristics. Supplementary Fig. 1 shows monthly incidence trends and seasonality. Analysis of COVID-19 cases relative to AMR pathogen cases did not indicate direct correlation (Supplementary Fig. 2).

Comparing sociodemographic characteristics (sex, age groups, federal states) before and during the first two pandemic years 2020–2021 revealed no substantial changes (Supplementary Figs. 3, 4 and Supplementary Table 1). The gender ratio of approximately 2/3 male cases, consistent with previous findings [22], remained unchanged during the pandemic. More than 75% of cases were older than 50 years in both periods.

For CRA, CRKP and CREC, we observed no statistically significant differences in the distribution of infection and colonisation between pre-pandemic and 2020–2021 pandemic periods (Supplementary Fig. 5 and Supplementary Table 1).

We found statistically nonsignificant increases in the number of deaths among infected CRA cases peaking between late 2020 and mid–2021, and for CRKP throughout 2021. Among MRSA cases, there were marginally, yet significantly fewer deaths documented towards by late 2020 (Supplementary Figs. 6, 7 and Supplementary Table 1).

**Fig. 1:**
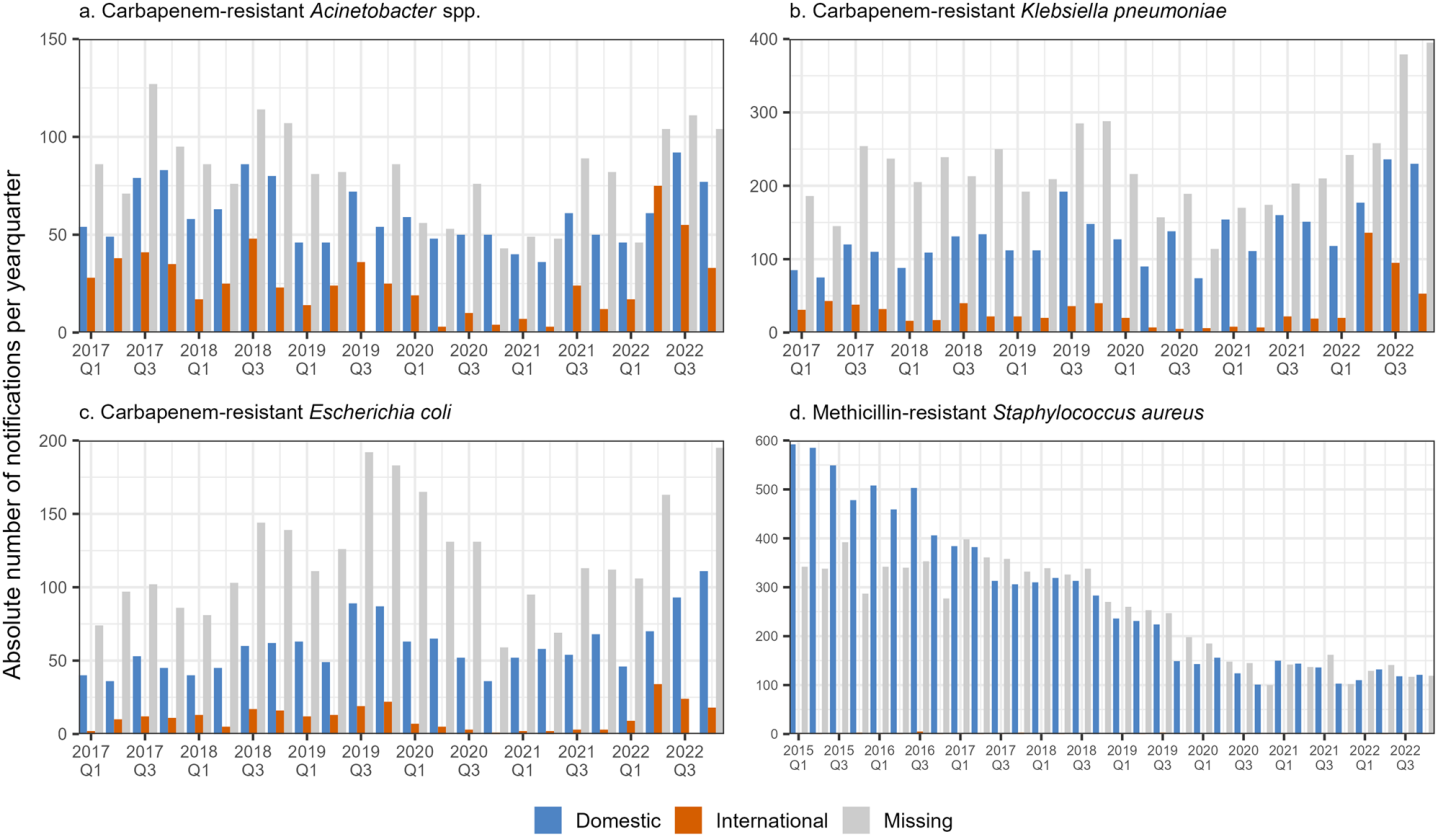
Statutory surveillance data – Absolute number of notifications by documented mobility history for statutory notifications of **a**. CRA (*n* = 4,079), **b**. CRKP (*n* = 9,347), **c**. CREC (*n* = 4,686) and **d**. MRSA (*n* = 17,090). Please note different y-axis scales. Please note the different time frame and reference definition for MRSA notifications, i.e. only including invasive infections. Germany, 2015–2022

International mobility (i.e. at least one international stay possibly relevant for exposure) decreased in 2020–2021 for CRA, CRKP and CREC. The subsequent 2022 increases were dominated by prior stays in Ukraine. Most reported international destinations were Ukraine, Türkiye^1^, Egypt and Greece (Fig. 1, Supplementary Table 1 and Supplementary Fig. 8). We found statistically significant differences in the distribution of presumed acquisition settings between both periods for CRA, CRKP and CREC. While patterns in nosocomial versus community settings remained similar, the decrease in presumed international acquisition during 2020–2021 may explain these statistical differences (Supplementary Table 1 and Supplementary Fig. 9).

**Table 1:** Statutory surveillance data – Characteristics of CRA (*n* = 4,079), CRKP (*n* = 9,347), CREC (*n* = 4,868) and invasive MRSA (*n* = 17,090) notifications. Percentages were calculated relative to all notifications with complete data for the respective variable. Please note the different time frame and reference definition for MRSA notifications, i.e. only including invasive infections. Germany, 2015–2022

Pathogen		CRA		CRKP		CREC		MRSA	
Characteristic		*n*	%	*n*	%	*n*	%	*n*	%
**Total**									
Total		4,079	100.0	9,347	100.0	4,686	100.0	17,090	100.0
**Sex**									
*Complete*		*4,074*	*100.0*	*9,309*	*100.0*	*4,671*	*100.0*	*17,049*	*100.0*
Female		1,356	33.3	3,347	36.0	2,081	44.5	5,991	35.1
Male		2,718	66.7	5,960	64.0	2,590	55.5	11,058	64.9
Diverse		0	0	2	0	0	0	0	0
**Age groups**									
*Complete*		*4,076*	*100.0*	*9,336*	*100.0*	*4,678*	*100.0*	*17,090*	*100.0*
0–18		167	4.1	527	5.6	404	8.6	910	5.3
19–49		864	21.2	1,690	18.1	957	20.5	1,242	7.3
50–69		1,547	38.0	3,232	34.6	1,586	33.9	5,186	30.3
> 70		1,498	36.8	3,887	41.6	1,731	37.0	9,752	57.1
**Infection / colonisation**									
*Complete*		*3,847*	*100.0*	*9,018*	*100.0*	*4,516*	*100.0*	*NA*	*NA*
Infected		1,951	50.7	4,179	46.3	1,833	40.6	NA	NA
Colonised		1,896	49.3	4,839	53.7	2,683	59.4	NA	NA
**Deaths (among infected cases)**									
*Complete*		*1,929*	*100.0*	*4,121*	*100.0*	*1,803*	*100.0*	*16,719*	*100.0*
Deceased		157	8.1	236	5.7	65	3.6	2,175	17.0
Not deceased		1,772	92.9	3,885	94.3	1,738	96.4	14,544	89.0
**Presumed acquisition setting**									
*Complete*		*3,776*	*100.0*	*8,690*	*100.0*	*4,270*	*100.0*	*NA*	*NA*
Probably nosocomial		718	19.0	2,008	23.1	689	16.1	NA	NA
Possibly nosocomial		1,838	48.7	4,709	54.2	2,516	58.9	NA	NA
Possibly community		644	17.1	1,264	14.5	832	19.5	NA	NA
Possibly international		576	15.3	709	8.2	233	5.5	NA	NA
**Mobility history**									
*Complete*		*2,056*	*100.0*	*3,937*	*100.0*	*1,700*	*100.0*	*9,112*	*100.0*
Domestic only		1,440	70.0	3,182	80.8	1,437	84.5	9,068	99.5
International		616	30.0	755	19.2	263	15.5	44	0.5
**Annually observed notifications**									
*Complete*		*4,079*		*9,347*		*4,686*		*17,090*	
Pre-pandemic	2015	NA		NA		NA		3,570	
	2016	NA		NA		NA		3,197	
	2017	786		1,356		568		2,843	
	2018	780		1,441		715		2,446	
	2019	711		1,653		955		1,838	
Pandemic	2020	480		1,169		739		1,126	
	2021	501		1,389		631		1,078	
	2022	821		2,339		1,078		992	

### Modelling statutory surveillance data

**Fig. 2:**
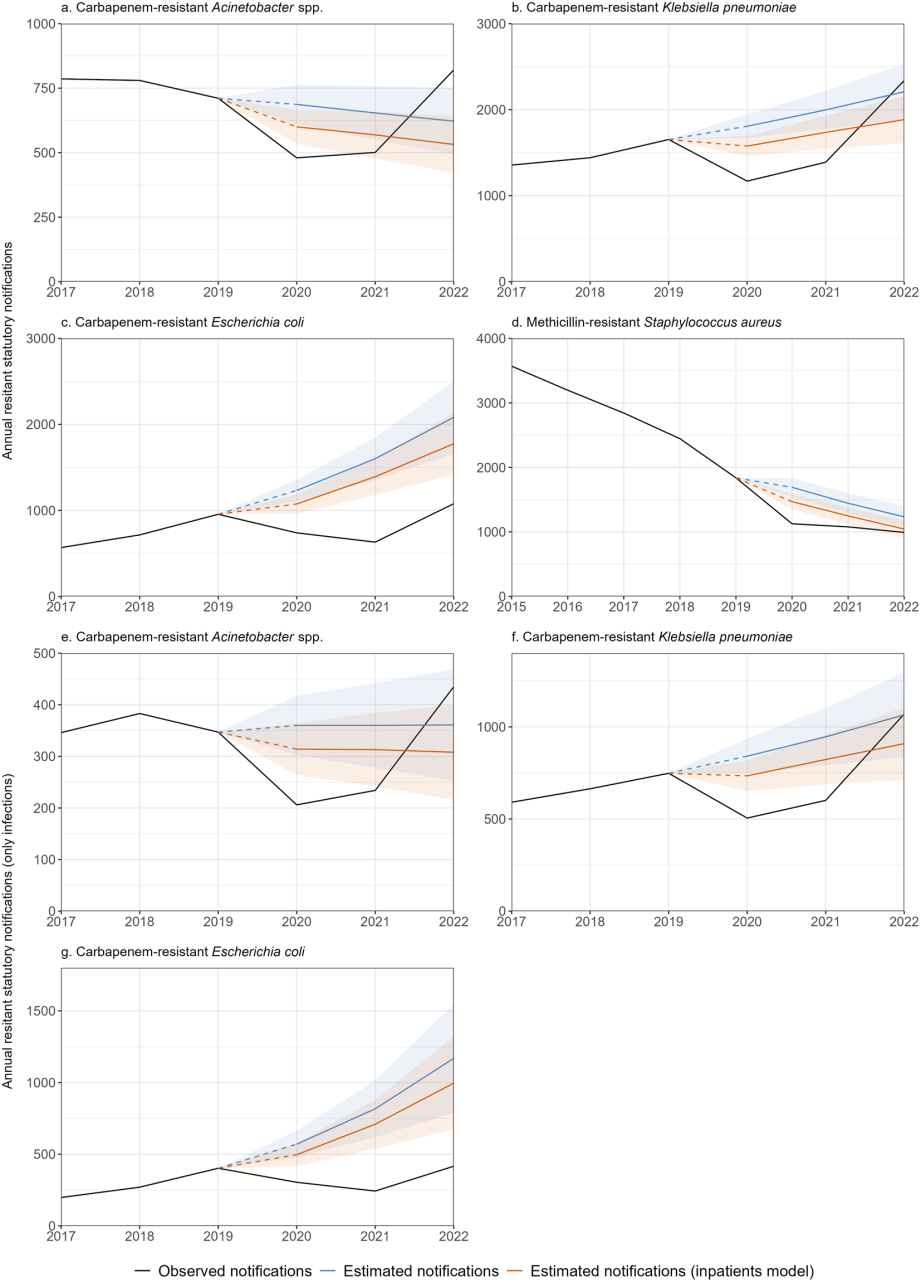
Statutory surveillance data – Results of annual models (blue) or models with inpatient offset (orange), i.e. comparison of hypothetically projected notification numbers, had the COVID-19 pandemic not occurred, to observed statutory notification numbers (black). **a**. CRA (*n* = 4,079), **b**. CRKP (*n* = 9,347) and **c**. CREC (*n* = 4,686) show notifications of infections and colonisations combined, while **d**. MRSA (*n* = 17,090), **e**. CRA (*n* = 1,951), **f**. CRKP (*n* = 4,179) and **g**. CREC (*n* = 1833) include only infections. Please note different y-axis scales. Please note the different time frame and statutory reference definition for MRSA notifications, i.e. only including invasive infections. Germany, 2015–2022

Modelling of annual CRA statutory surveillance data (both infected and colonised) without consideration of inpatient counts revealed statistically significant reductions in observed notifications relative to hypothetical projections. These were − 30% (95%CI: -39%|-20%) in 2020 and − 23% (-36%|-8%) in 2021, followed by an increase above projections with + 32% (+ 6%|+64%) in 2022. CRKP showed similar trends with − 35% (-41%|-29%) and − 31% (-38%|-22%) reductions in 2020–2021, and a subsequent + 6% (-9%|+23%) increase above hypothetical projections in 2022. CREC demonstrated reductions of -40% (-47%|-32%) and − 61% (-67%|-53%) in 2020–2021. Despite an increase in 2022, we still observed − 48% (-58%|-36%) fewer notifications relative to projections. After falling in 2020 MRSA notifications levelled at a plateau. Relative to hypothetical projections of the pre-pandemic falling trend, this resulted in -33% (-41%|-25%), -25% (-35%|-14%) and − 20% (-32%|-6%) reductions. Reductions in 2020–2021 were equivalent to 360 CRA, 1246 CRKP, 1464 CREC and 931 MRSA cases fewer than projected (Fig. 2, Supplementary Tables 2 and 3).

We observed similar trends when considering inpatient counts. Due to relatively lower hypothetical projections of annual incidence (compared to the above model), reductions were generally smaller and increases more pronounced (Fig. 2, Supplementary Tables 2 and 3). Modelling only infected CRA, CRKP and CREC case notifications showed trends to those combining infections and colonisations (Fig. 2, Supplementary Tables 2 and 3).

Our weekly model represented seasonality and sub-annual developments better, yet overall trends concurred with previous annual models (Supplementary Fig. 10, Supplementary Tables 2 and 3).

### Modelling antibiotic resistance surveillance data (ARS)

**Fig. 3:**
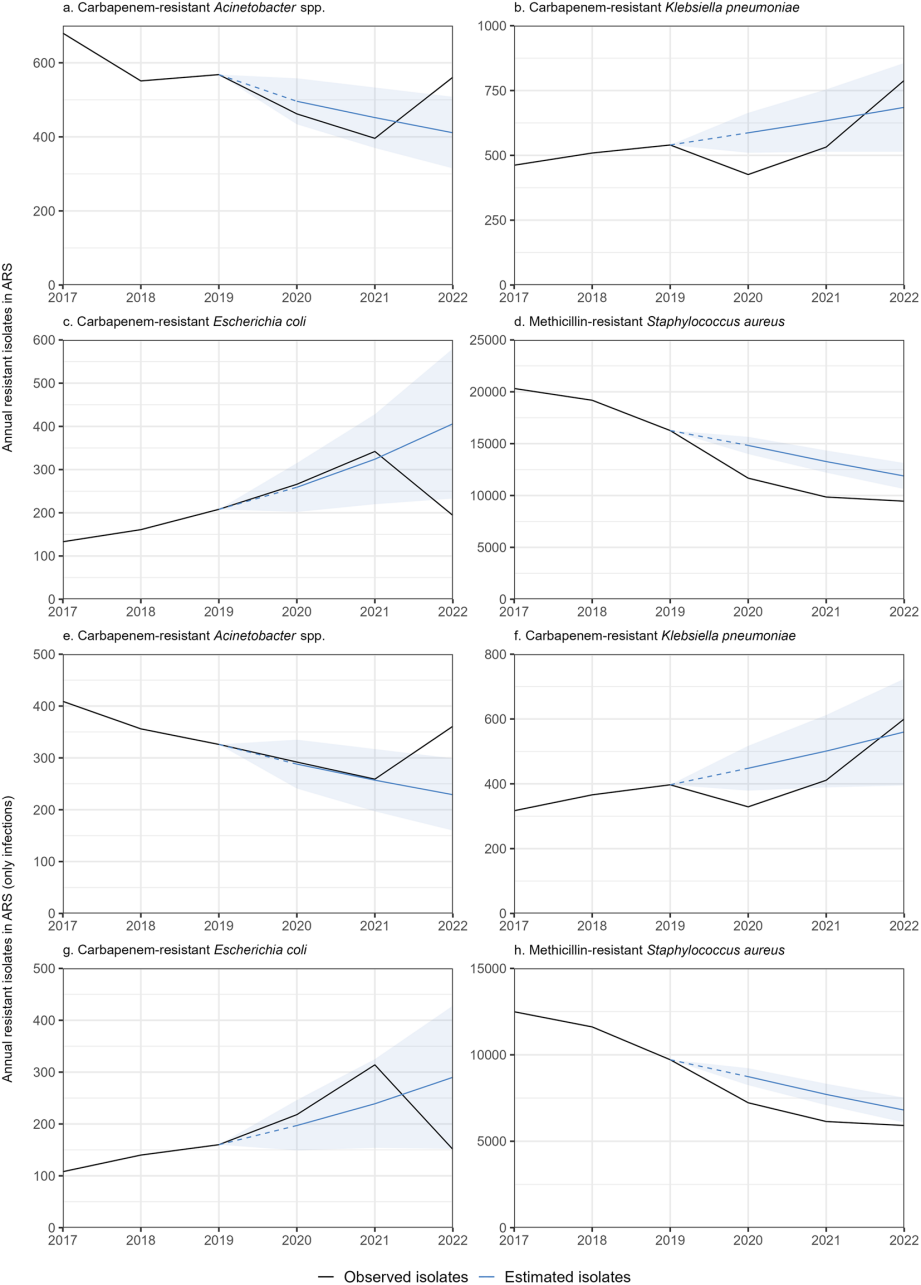
Antibiotic Resistance Surveillance (ARS) data – Results of annual models for all resistant isolates in ARS, i.e. comparison of observed isolate numbers (black) and hypothetically projected isolate numbers, had the COVID-19 pandemic not occurred (blue). **a**. CRA. (*n* = 3,218), **b**. CRKP (*n* = 3,258), **c**. CREC (*n* = 1,304) and **d**. MRSA (*n* = 86,752) show all (i.e. infections and colonisations), while **e**. CRA. (*n* = 2,003), **f**. CRKP (*n* = 2,420), **g** CREC (*n* = 1,091) and **h** MRSA (*n* = 53,108) include only infections based on sample material. Please note different y-axis scales. Germany, 2017–2022

From ARS we included *n* = 3,218 CRA, *n* = 3,258 CRKP, *n* = 1,304 CREC, and *n* = 86,752 MRSA isolates (Table 2), whose modelling results are summarized in Fig. 3, Supplementary Tables 4 and 5. CRA demonstrated reductions of -7% (-20%|+9%) in 2020 and − 12% (-29%|+8%) in 2021 relative to hypothetical projections, followed by + 36% (+ 29%|+75%) increase in 2022. Changes were − 27% (-38%|-15%), -16% (-32%|+3%) and + 15% (-11%|+49%) for CRKP, and − 21% (-26%|-16%), -26% (-32%|-19%) and − 20% (-19%|-11%) for MRSA. While the annual changes differed, overall these pathogen trends were relatively similar to the statutory models, particularly for MRSA. CREC showed opposite trends with + 3% (-20%|+32%) and + 6% (-25%|+48%) increases and a subsequent − 52% (-70%|-25%) reduction.

Considering infections only (based on sample material), we generally observed similar trends compared to the ARS-based models combining infections and colonisations (Table 2; Fig. 3, Supplementary Tables 4 and 5).

To better reflect the outpatient sector, we stratified resistant isolates by inpatient and outpatient care type. CRA, CRKB and MRSA demonstrated reductions in 2020–2021, followed by increases (or smaller reductions) in 2022. Overall trends for both outpatients and inpatients concur with statutory models, except for CREC. However, annual isolate numbers, apart from MRSA, were relatively low, and results mostly statistically insignificant (Supplementary Figs. 11, 12, Supplementary Tables 4 and 5).

We also modelled overall ARS trends for all isolates (resistant and non-resistant) and the proportion of resistant isolates (Supplementary Figs. 13, 14, Supplementary Tables 4 and 5). We observed reductions for all pathogens in all three pandemic years compared to hypothetical projections.

**Table 2:** Antibiotic Resistance Surveillance (ARS) data – Characteristics of CRA (*n* = 3,218), CRKP (*n* = 3,258), CREC (*n* = 1,304) and MRSA (*n* = 86,752) isolates in ARS. Percentages were calculated relative to all notifications with complete data for respective variables. Germany, 2017–2022

Pathogen		CRA		CRKP		CREC		MRSA	
Characteristic		*n*	%	*N*	%	*n*	%	*n*	%
**Total**									
Total		3,218	100.0	3,258	100.0	1,304	100.0	86,752	100.0
**Sex**									
*Complete*		*2,730*	*100.0*	*2,627*	*100.0*	*946*	*100.0*	*70,046*	*100.0*
Female		891	32.6	931	35.4	496	52.4	27,848	39.8
Male		1,839	67.4	1,696	64.6	450	47.6	42,198	60.2
**Age groups**									
*Complete*		*3,218*	*100*	*3,258*	*100*	*1,304*	*100*	*86,752*	*100*
0–18		179	5.6	121	3.7	69	5.3	6,767	7.8
19–49		540	16.8	590	18.1	160	12.3	13,653	15.7
50–69		1180	36.7	1220	37.4	373	28.6	24,413	28.1
> 70		1319	41.0	1327	40.7	702	53.8	41,919	48.3
**In-/outpatient care type**									
*Complete*		*3,218*	*100.0*	*3,257*	*100.0*	*1,304*	*100.0*	*86,751*	*100.0*
Outpatient		648	20.1	303	9.3	325	24.9	26,903	31.0
Inpatient		2,570	79.9	2,954	90.7	979	75.1	59,848	69.0
**Infection / colonisation (based on sample material)**									
*Complete*		*3,218*	*100.0*	3,258	*100.0*	*1,304*	*100.0*	86,752	*100.0*
Infection		2,003	62.2	2,420	74.3	1,091	83.7	53,108	61.2
Colonisation		1,215	37.8	838	25.7	213	16.3	33,644	38.8
**Annual observed isolates**									
*Complete*		*3,218*		*3,258*		*1,304*		*86,752*	
Pre-pandemic	2017	680		462		133		20,320	
	2018	551		509		161		19,185	
	2019	568		540		208		16,266	
Pandemic	2020	462		426		266		11,673	
	2021	396		532		342		9,854	
	2022	561		789		194		9,454	

## Discussion

### Summary of results

We described and quantified changes in the epidemiology of CRA, CRKP, CREC and MRSA in Germany before (2015/2017–2019) and during the COVID-19 pandemic (2020–2022), using data from two surveillance systems.

The mix of cases by sex, age and infection/colonisation did not generally differ between the pre-pandemic and pandemic periods. We observed a disproportionate reduction of cases with prior stays in foreign countries during 2020–2021, followed by a rebound in 2022. Modelling statutory data revealed decreases in notifications with CRA, CRKP, CREC infections and colonisations in 2020–2021 relative to projections without the pandemic, followed by increases in 2022. MRSA infections fell below hypothetical projections in all three pandemic years. When accounting for concurrent inpatient reductions, trends remained similar for all pathogens, although relative to overall lower projections. Models including only CRA, CRKP and CREC infections mirrored combined infected and colonised models. Weekly modelling with seasonality corroborated first annual model findings.

Resistant pathogen isolates from ARS showed analogous trends for CRA, CRKP and MRSA. However, resistant CREC isolates increased in 2020–2021 and decreased in 2022 relative to hypothetical projections. Models based on resistant isolates from infections only (based on sample material) aligned with those including all resistant isolates. In- and outpatient isolate numbers were relatively low, with most results not statistically significant, yet overall trends support statutory findings, except for CREC.

### Interpretation of observed trends and possible hypotheses

A combination of factors likely influenced the observed development of AMR pathogens. In our annual statutory model, we observed statistically significant reductions of CRA, CRKP, CREC and MRSA notified cases in 2020–2021 compared to hypothetical projections. This suggests a reduction of AMR pathogen infections and colonisations during the pandemic. However, fewer medical procedures and non-COVID-19 patients may have contributed to fewer nosocomial transmission [23]. Our second model accounted for the concurrent inpatient reduction [18]. We still observed reductions in 2020–2021 for all pathogens, yet with lower amplitude. Fewer inpatients likely contributed to reductions in AMR pathogen incidence, yet we observed a further reduction beyond this effect. Since this analysis neglects the pandemic impact on the outpatient sector, we modelled resistant isolates in ARS stratified by in- and outpatient. Generally, outpatient trends concurred with the inpatient sector, indicating a similar impact on the outpatient sector. Apart from MRSA, annual numbers in ARS were relatively low and mostly non-significant, warranting cautious interpretation.

Another possible explanation for observed reductions could be underdetection in the statutory surveillance through underreporting or less screening and testing [24]. Asymptomatic colonised patients are predominantly found through hospital screenings [25]. Due to fewer screenings during the pandemic, fewer asymptomatic cases could be detected and reported. Conversely, (severely) symptomatic infected patients presumably have a lower risk of underdetection. In our regression including only infected patients, we observed statistically significant reductions for infected case notifications for all pathogens in 2020–2021. While we cannot exclude underreporting, comparable trends in the statutory and laboratory surveillance indicate it was not a major contributing factor. Additionally, we observed statistically significant reductions in all three pandemic years when analysing all (resistant and non-resistant) isolates in ARS.

Studies reported coinfections of AMR pathogens with COVID-19 [26], potentially exacerbated by (over)use of antibiotics [27]. Together with increasing health-care associated transmissions [28] and personnel shortages [23] these factors could facilitate increasing AMR pathogen trends and higher mortality. We observed non-significant increases of deaths among infected case notifications with CRA in 2020 and CRKP in 2021 and a statistically significant reduction for MRSA in 2021. Notably, we found no increase of “probable” or “possible nosocomial” acquisition relative to notification numbers, nor a correlation of AMR pathogen trends with COVID-19 waves as may be expected for coinfections.

AMR pathogen reductions in 2020–2021 may also be explained by changes in risk factors due to effects of non-pharmaceutical interventions [24]. While non-pharmaceutical interventions mainly aimed at respiratory pathogen transmission, they plausibly also affected AMR pathogen transmission [29, 30]. Fewer patient transfers, social distancing and behavioural changes may have reduced person-to-person transmission [31]. In 2022 CRA, CRKP, and CREC notifications increased relative to hypothetical projections without the pandemic. MRSA showed a smaller reduction in 2022, which, considering the pre-pandemic falling trend, may still reflect a changing trend for MRSA. The 2022 increases could be linked to the gradual lifting of COVID-19 related public health measures or changes in social distancing. They could also indicate the recurrence of pre-pandemic rising trends, particularly for CRKP and CREC.

International mobility is another risk factor for AMR infections [12, 29]. In 2020–2021 we found a statistically significant decrease in international mobility of cases, likely contributing to observed reductions. Contrarily, in 2022 we observed an increase of international mobility for CRA, CRKP, and CREC cases. Touristic destinations like Türkiye^2^, Egypt, and Greece were most reported. These countries report high AMR incidences, which increased during the pandemic [32–34]. Local AMR challenges are discussed to be attributed to inadequate consumption of antibiotics, partially unregulated access and limited community awareness [34–36]. In 2022, the observed increase was primarily associated with refugees and medically evacuated patients from Ukraine following the Russian invasion. Rates of AMR are high in Ukraine and have surged during the conflict [37]. Increases in NDM-1 and NDM-1/OXA-48-producing CRKP in Germany in 2022, associated with the war in Ukraine, have been reported [38]. This indicates that international mobility may have contributed to 2022 increases, rather than COVID-19 related effects alone.

Internationally, AMR pathogen trends appear to depend on the context and local underlying factors. The United States of America reported increases for CRA (+ 35%), drug-resistant Enterobacterales (+ 10%) and invasive hospital-onset MRSA (+ 13%) from 2019 to 2020 [39]. A more recent publication suggests more divergent trends of AMR in the US during the pandemic [40]. In the European Union and European Economic Area (EU/EEA), between 2017 and 2021, percentages of resistant isolates for MRSA were stable or declining, while increasing for CRA, CREC and CRKP. Large inter-country variations were observed, often along a north-to-south and west-to-east gradient of resistance [32]. A recent study on bloodstream CRA infections in the EU/EEA found the largest increases in countries that already had high percentages of resistant isolates prior to 2020 [41]. International mobility, particularly to high incidence countries, may partially explain CRA, CREC and CRKP increases in Germany.

### Limitations

The following limitations should be considered:

- Our study predicts hypothetical trends, but may not identify singular driving factors nor establish causality. By assessing different models and combining two surveillance sources, we can postulate hypotheses regarding underlying factors and assess if observed changes reflect real pathogen trends or if they were influenced by factors like fewer inpatients or underreporting.
- Annual models simplify pathogen seasonality and sub-annual effects, yet they adequately describe overall trends. Moreover, weekly sinusoidal modelling with seasonality showed comparable trends.
- The relatively short study period, particularly pre-pandemic, may distort trends and affects the robustness of regression over time.
- We used hospital inpatient numbers as proxy for overall changes in patient numbers, neglecting the outpatient sector, where pandemic effects may differ. Stratification by in- and outpatient in ARS partially addressed this, yet more research regarding the outpatient sector is needed.
- Data quality and completeness, particularly for mobility history, was limited and declined further during the pandemic, possibly due to workload.
- Due to a lack of data regarding comorbidities and previous SARS-CoV-2 infections, we could not assess their relevance on the host susceptibility for AMR infections. This may be an important topic for future studies.
- ARS coverage depends on voluntarily participating laboratories and may not be representative for Germany.

## Conclusion

Our study described and quantified changes in CRA, CRKP, CREC and MRSA epidemiology in Germany before and during the COVID-19 pandemic. We observed statistically significant reductions in statutory AMR notification incidences in 2020–2021, followed by increases for CRA, CRKP and CREC in 2022. Even after accounting for fewer hospital inpatients, these reductions persisted. Moreover, both statutory and laboratory-based ARS data showed similar trends, implying a genuine reduction in AMR colonisations and infections during the pandemic in Germany.

Contributing factors likely include fewer hospitalisations and reduced international mobility. Rising international mobility may partially explain the observed increases for CRA, CRKP and CREC in 2022. This underscores the importance of patient screening upon admission to healthcare facilities according to national guidelines, particularly for patients with previous contact to health systems in countries with high AMR pathogen prevalence [42].

The possibility of AMR pathogen reductions offers hope in combatting AMR, yet further research is needed to elucidate underlying factors and identify effective infection prevention and control strategies. Our study supports this by providing a better understanding of AMR epidemiology in Germany. Even amidst competing health crises, AMR surveillance must continue.

## Electronic supplementary material

Below is the link to the electronic supplementary material.

Supplementary Material 1

## Data Availability

The datasets used and/or analysed during the current study (weekly aggregated data), as well as the analytical code scripts, are available from the corresponding author on reasonable request.

## Footnotes

1 English nomenclature was based on the United Nations convention for the Republic of Türkiye.

2 English nomenclature was based on the United Nations convention for the Republic of Türkiye.
