# Supplementary material for: Changes in incidence and epidemiology of antimicrobial resistant pathogens before and during the COVID-19 pandemic in Germany, 2015–2022
Source: BMC Microbiol. 2025 Jan 28;25:51. doi: 10.1186/s12866-024-03723-5 (PMC11773696; doi:10.1186/s12866-024-03723-5)
Supplement: Supplementary file 1 — Supplementary Material 1 [file 12866_2024_3723_MOESM1_ESM.docx]

**Supplementary Material**

Changes in incidence and epidemiology of antimicrobial resistant pathogens before and during the COVID-19 pandemic in Germany, 2015 – 2022

*Supplementary Text 1: Further details regarding the statistical models*

We first fitted Poisson or negative binomial regression models per pathogen to the notification numbers in the statutory surveillance system per year throughout the study period (from 2015 for MRSA and 2017 for the other pathogens until 2022). We included a dummy covariate that was 0 for pre-pandemic and 1, 2 or 3 for the three pandemic years (2020–2022). We selected Poisson or negative binomial regression based on likelihood-ratio-test.

*Model input structure: Case numbers ~ Year + COVID_dummy*

We predicted hypothetical notification numbers had the pandemic not occurred. For that purpose, we predicted case numbers for 2020-2022 with the dummy covariate set to 0 for all years.

In a second iteration, we repeated both steps above (model fit and counterfactual model) complemented with hospital inpatient counts as offset.

*Model input structure: Case numbers ~ Year + COVID_dummy + offset[log(inpatients)]*

For Antibiotic Resistance Surveillance (ARS), we repeated the steps above but varied the outcome by including instead of annual notification case numbers: all isolates, resistant isolates, resistant isolates from infections only, as well as resistant isolates stratified by inpatient or outpatient care type.

*Model input structure: Case numbers ~ Year + COVID_dummy*


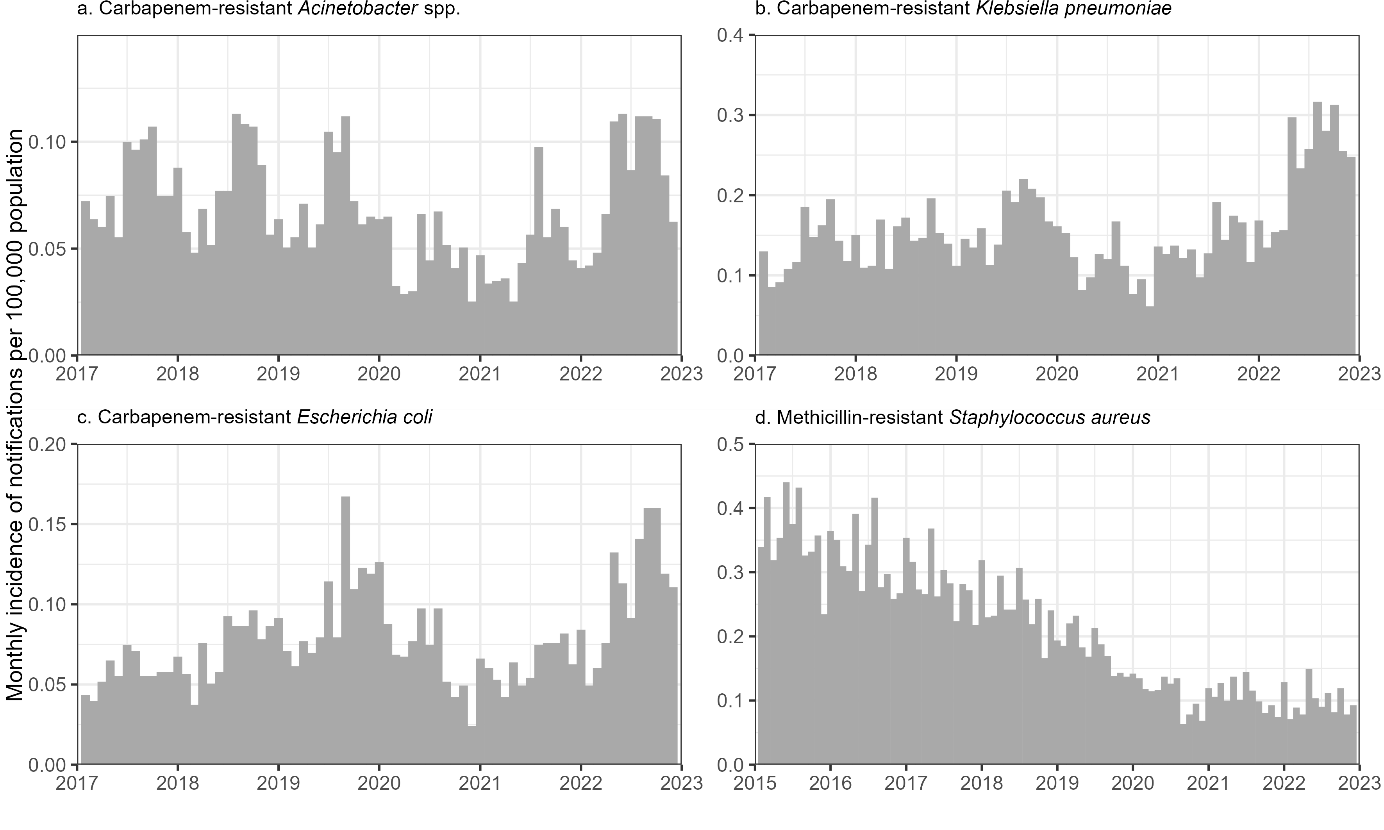


Supplementary Figure 1: Statutory surveillance data – Monthly incidences per 100,000 population over time for a. CRA (n = 4,079), b. CRKP (n = 9,347), d. CREC (n = 4,686) and d. MRSA (n = 17,090). Please note the different y-axis scales. Please note the different time frame and reference definition for MRSA notifications, i.e. only including invasive infections. Germany, 2015 – 2022.


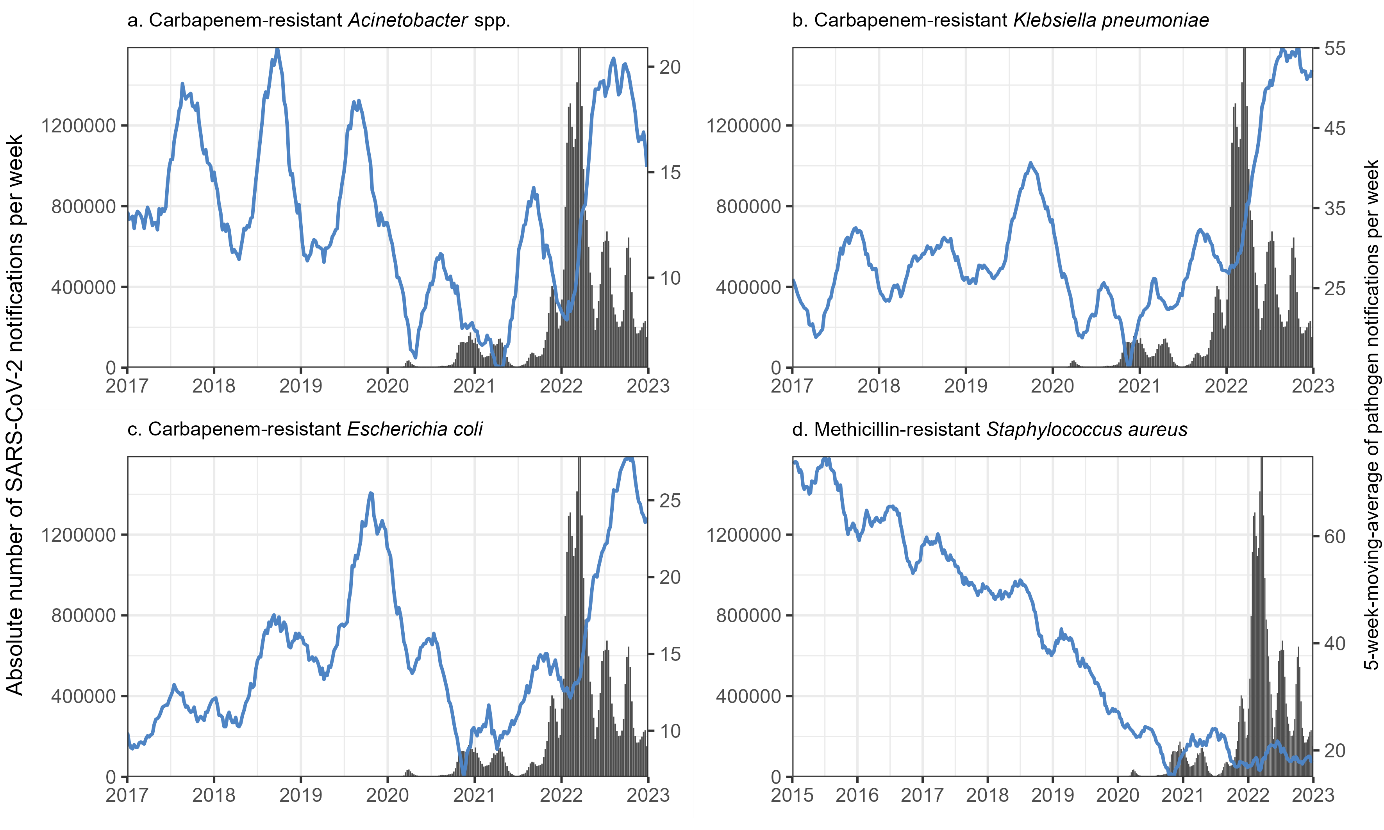


Supplementary Figure 2: Statutory surveillance data – Comparison of trends of weekly statutory Sars-CoV-2 (n = 37,394,380) notifications (black) with weekly statutory notifications (blue) of a. CRA (n = 4,079), b. CRKP (n = 9,347), c. CREC (n = 4,686) and d. MRSA (n = 17,090). Please note the different y-axis scales. Please note the different time frame and reference definition for MRSA notifications, i.e. only including invasive infections. Germany, 2015 – 2022.


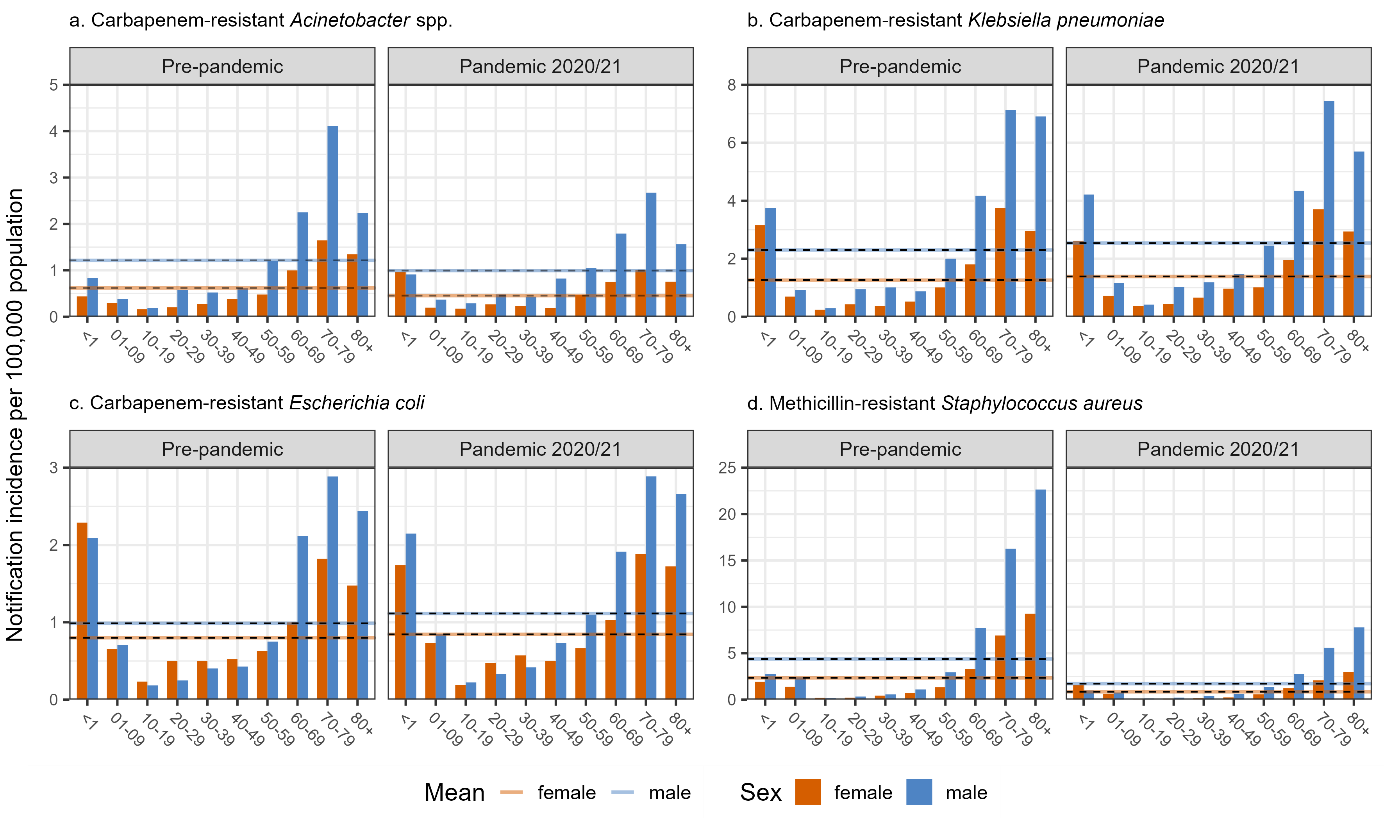


Supplementary Figure 3: Statutory surveillance data – Incidences per 100,000 population by sex and age groups before and during the pandemic years 2020 – 2021 and mean incidences per period for a. CRA (n = 4,074), b. CRKP (n = 9,309), c. CREC (n = 4,671) and d. MRSA (n = 17,049). Diverse sex category is not shown (n=2). Please note the different y-axis scales. Please note the different time frame and reference definition for MRSA notifications, i.e. only including invasive infections. Germany, 2015 – 2021.


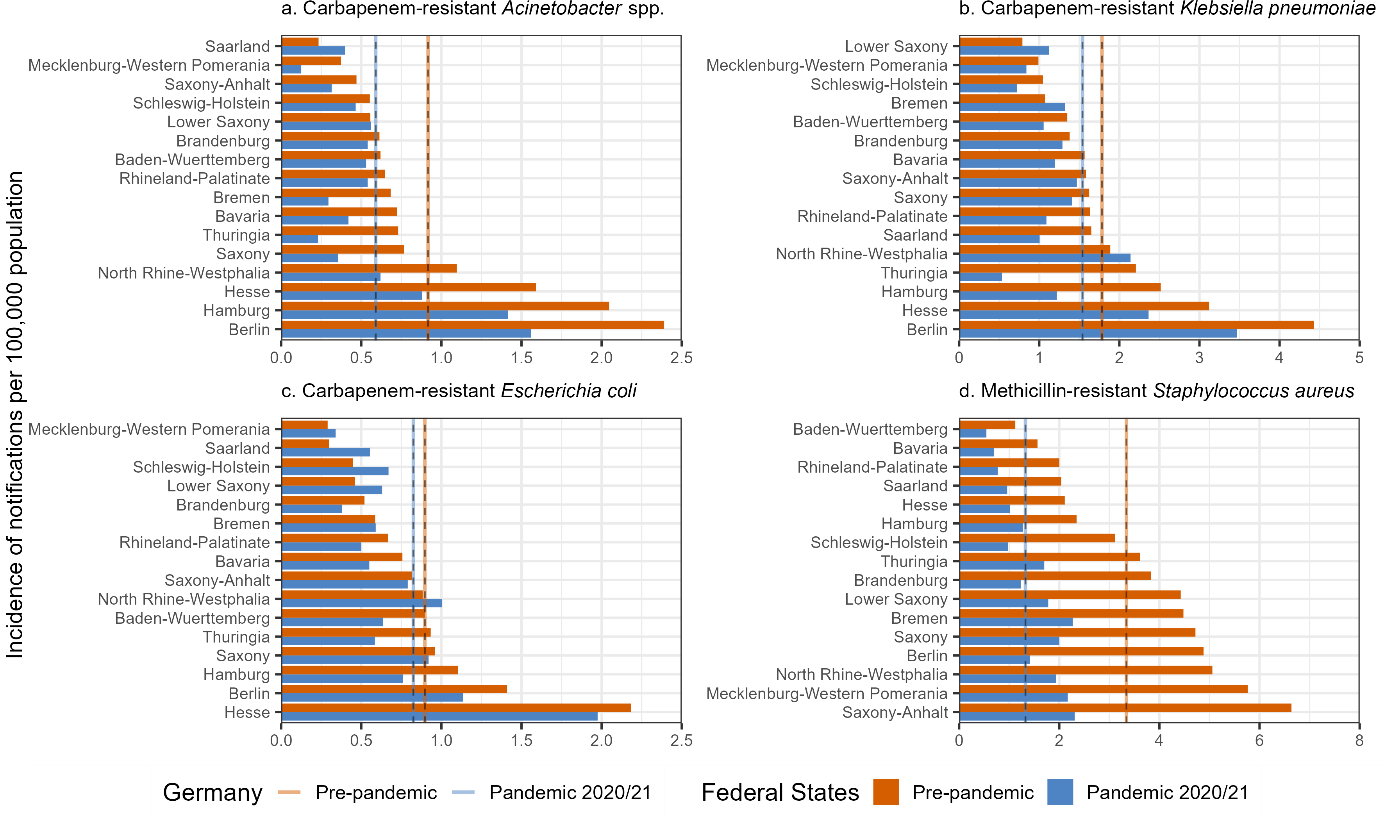


Supplementary Figure 4: Statutory surveillance data – Notification incidences per (notifying) German federal state and overall Germany before and during pandemic years 2020 – 2021 for a. CRA (n = 4,708), b. CRKP (n = 9,344), c. CREC (n = 4,683) and d. MRSA (n = 17,077). Please note the different y-axis scales. Please note the different time frame and reference definition for MRSA notifications, i.e. only including invasive infections. Germany, 2015 – 2021.

Supplementary Table 1: Statutory surveillance data – Results of statistical chi-squared test for differences in the distribution of variables in pre-pandemic period and the first two pandemic years 2020 – 2021 for statutory CRA (n = 4,079), CRKP (n = 9,347), CREC (n = 4,868) and MRSA (n = 17,090) notifications. Please note the different time frame and reference definition for MRSA notifications, i.e. only including invasive infections. Germany, 2015 – 2021.

| **Distribution of variable** | ***p*-values** | | | |
| --- | --- | --- | --- | --- |
|  | **CRA** | **CRKP** | **CREC** | **MRSA** |
| **Sex** | Not significant | Not significant | Not significant | Not significant |
| **Age groups** | 0.015 | < 0.001 | Not significant | < 0.001 |
| **Infection / colonisation** | Not significant | Not significant | Not significant | NA |
| **Suspected nosocomial exposure** | < 0.001 | < 0.001 | < 0.001 | NA |
| **Outcome deaths (among infected)** | Not significant | Not significant | Not significant | < 0.001 |
| **Suspected exposure location** | < 0.001 | < 0.001 | < 0.001 | NA |


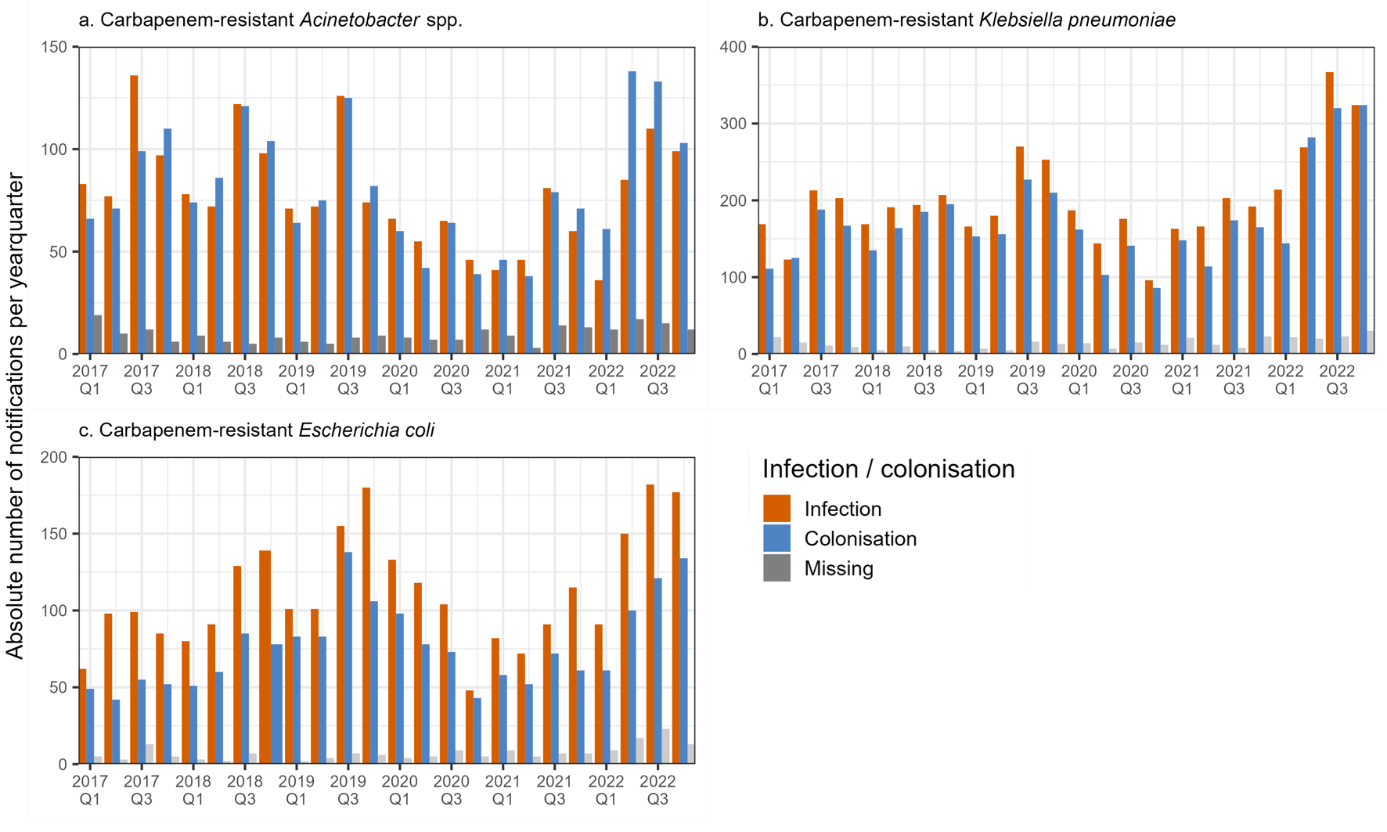


Supplementary Figure 5: Statutory surveillance data – Infection/colonisation status for statutory notifications per yearquarter of a. CRA (n = 4,079), b. CRKP (n = 9,347), c. CREC (n = 4,686). The variable was derived from the respective statutory variable complemented with sample material. Please note the different y-axis scales. Germany, 2015 – 2022.


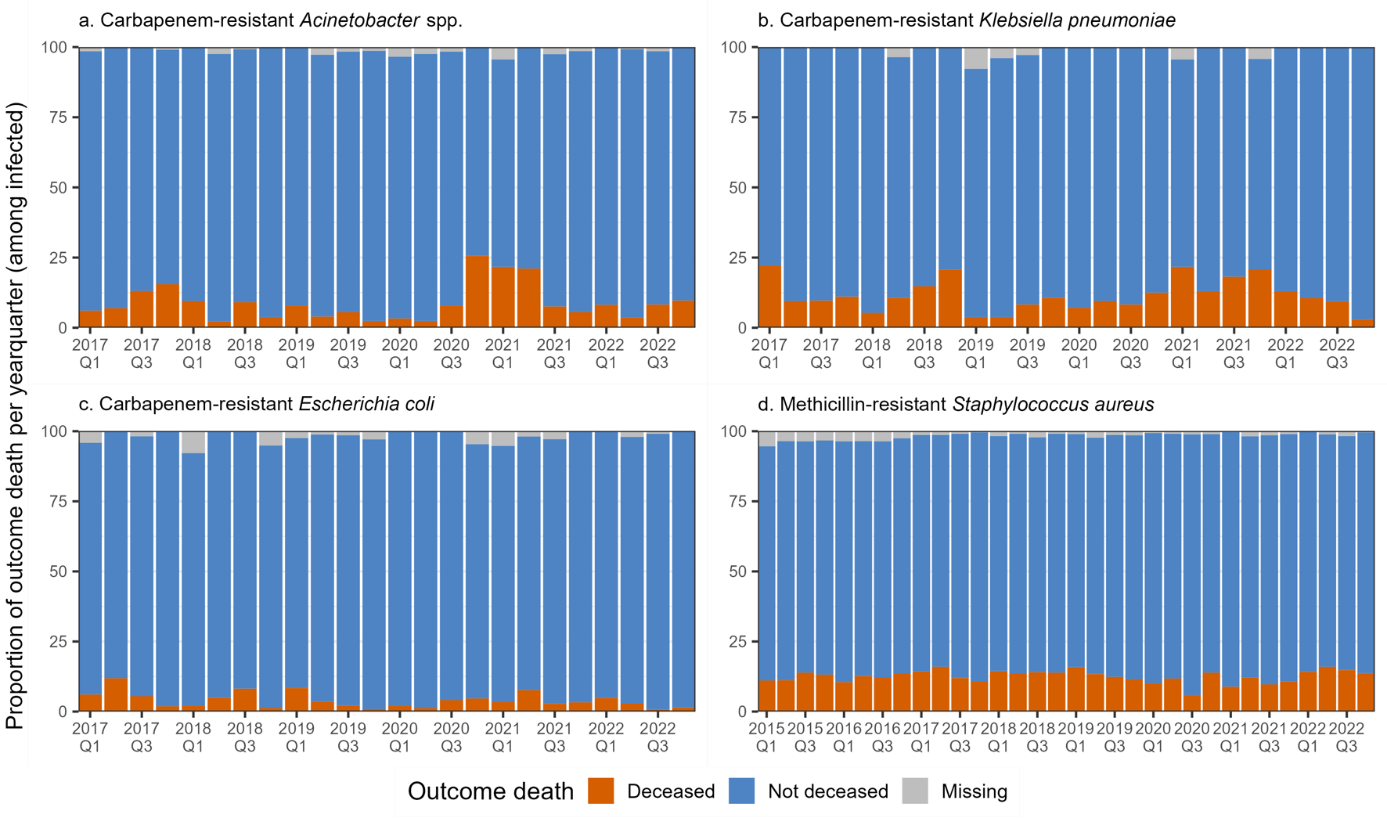


Supplementary Figure 6: Statutory surveillance data – Proportions of outcome death per yearquarter among infected cases for a. CRA (n = 1,951), b. CRKP (n= 4,179), b. CREC (n = 1,833) and d. MRSA (n = 17,090) notifications. Please note the different timeframe for MRSA. Germany, 2015 – 2022.


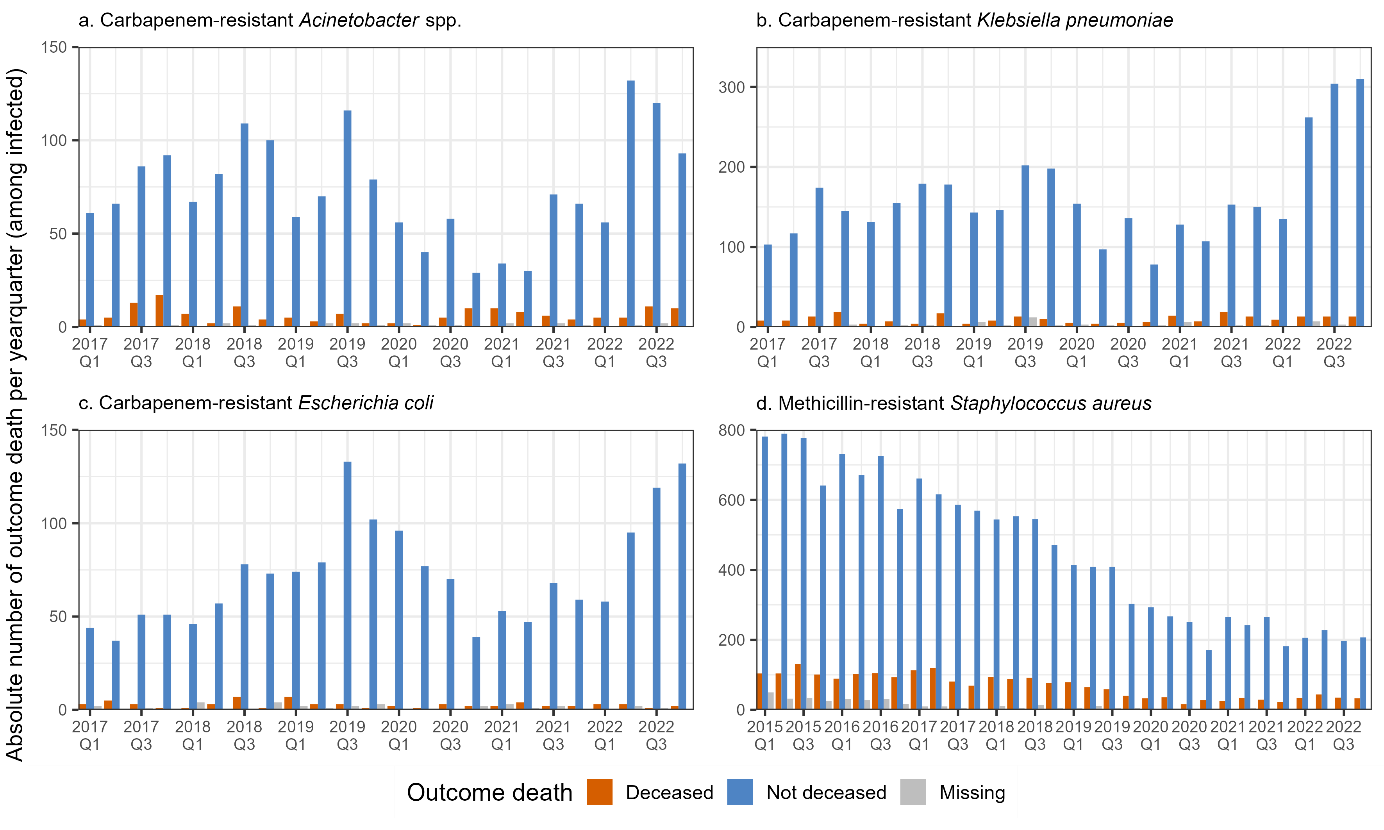


Supplementary Figure 7: Statutory surveillance data – Absolute number of outcome death per yearquarter among infected case notifications for a. CRA (n = 1,951), b. CRKP (n= 4,179), b. CREC (n = 1,833) and d. MRSA (n = 17,090) notifications. Please note the different y-axis scales and timeframe for MRSA. Germany, 2015 – 2022.


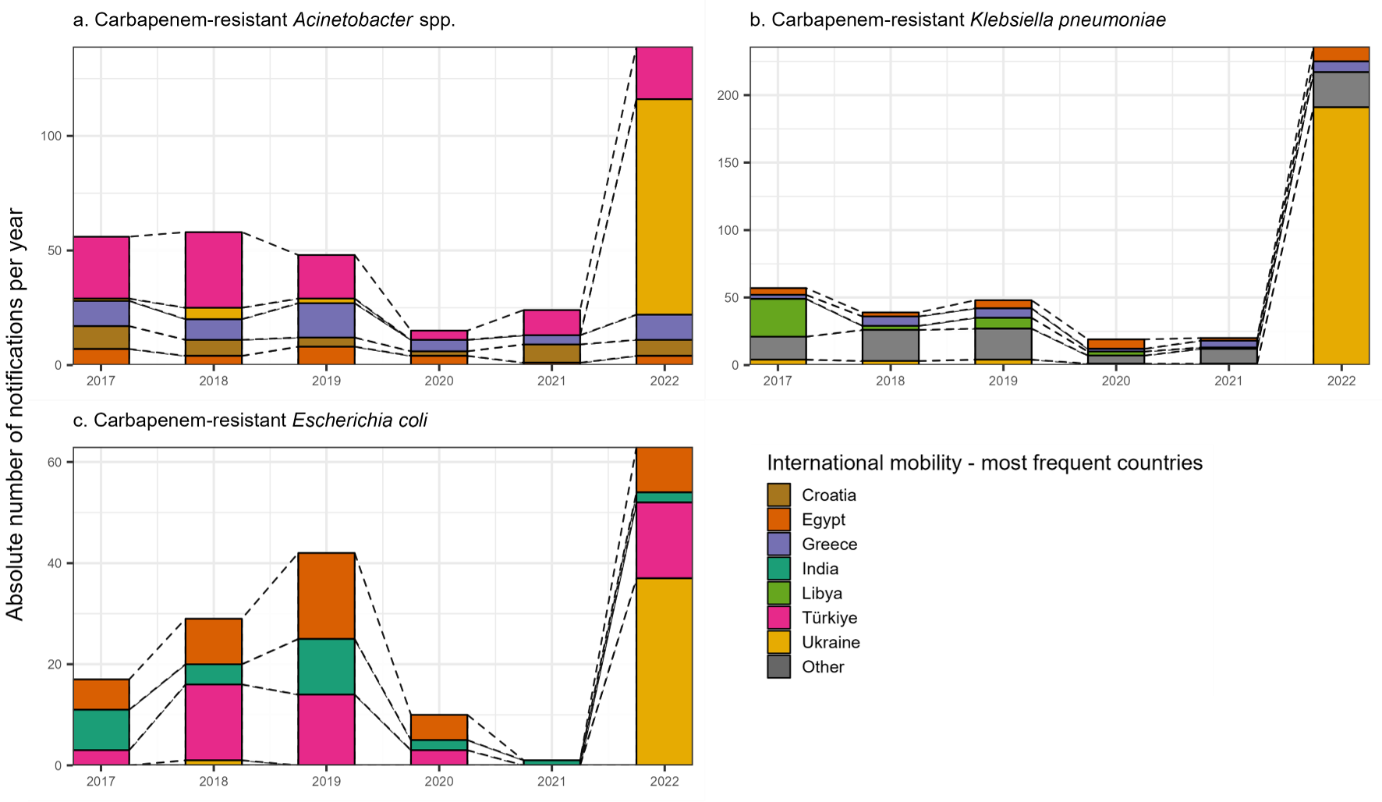


Supplementary Figure 8: International mobility – Most frequently reported countries (i.e. at least one international stay possibly relevant for exposure) per year for statutory notifications of a. CRA (n = 608), b. CRKP (n = 756) and c. CREC (n = 268). Per pathogen, the top five countries with highest reported numbers overall were included. For CRKP less frequent countries were grouped as “other”. We excluded MRSA due to low overall documented international mobility history. Please note the different y-axis scales. Germany, 2017 – 2022.


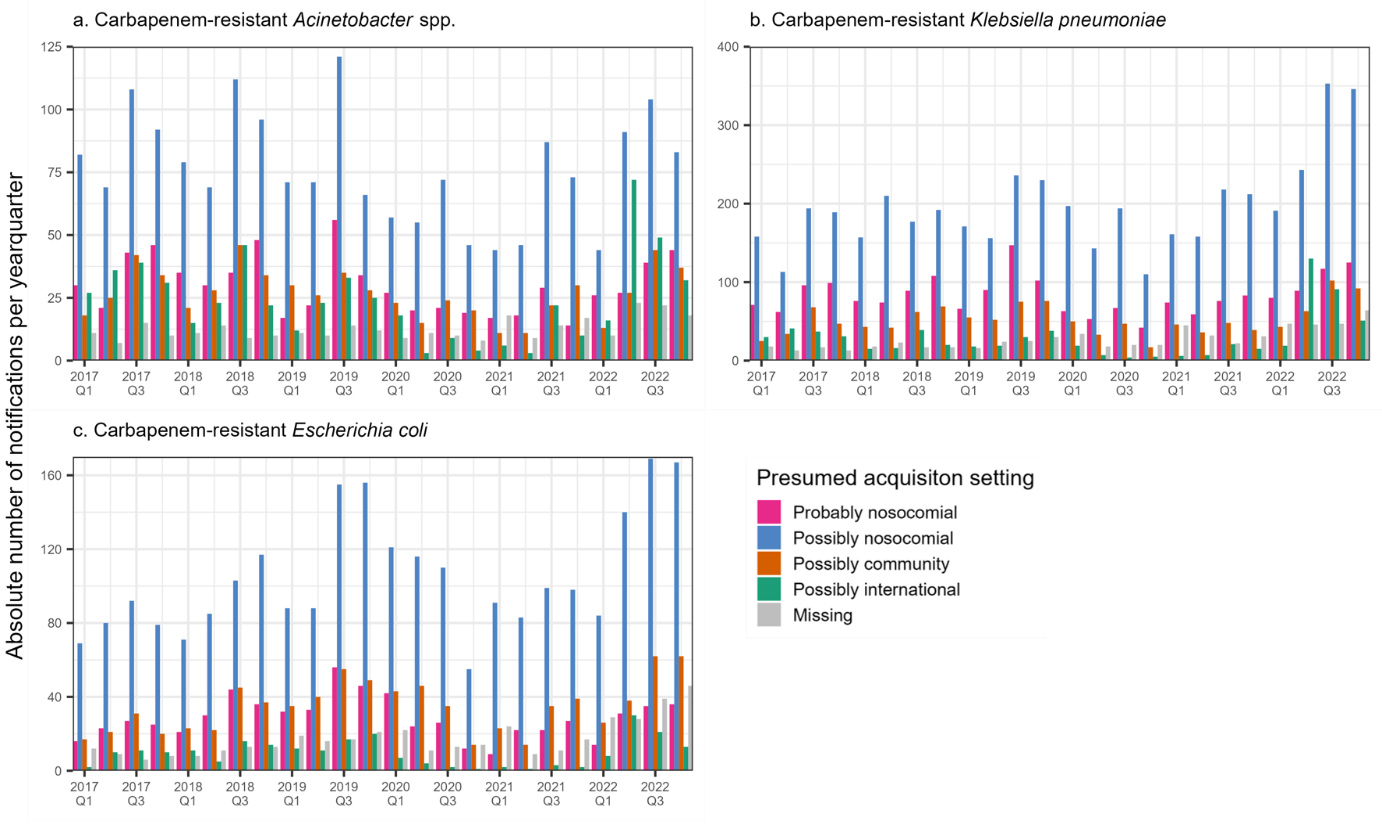


Supplementary Figure 9: Statutory surveillance data – Presumed acquisition setting for statutory notifications of a. CRA (n = 4,079), b. CRKP (n = 9,347), c. CREC (n = 4,686). The variable was defined based a combination of statutory variables including infection/colonisation, (international) mobility history, hospitalisation date(s), sample date(s), sample material(s), outbreak involvement and outbreak setting. Please note the different y-axis scales. Germany, 2015 – 2022.

Supplementary Table 2: Statutory surveillance data – Results of annual models, only infected models and weekly models with seasonality, each without and with inpatient offset, for statutory notifications of CRA, CRKP, CREC and MRSA. Incidence rate ratios were interpreted as relative changes (Δ) per pandemic year, i.e. as relative reductions (negative values) or increases (positive values) of observed annual notification numbers relative to hypothetically projected notification numbers without the COVID-19 pandemic. Statistical significance indicated as 95% confidence intervals (95%CI) and p-values (p). Germany, 2017 – 2022.

| **Infected and colonised** | | | | | | | | | | | | | | | | |
| --- | --- | --- | --- | --- | --- | --- | --- | --- | --- | --- | --- | --- | --- | --- | --- | --- |
| **Annual model without inpatient count** | | | | | | | | | | | | | | | | |
|  | **CRA (*n* = 4,079)** | | | | **CRKP (*n* = 9,347)** | | | | **CREC (*n* = 4,686)** | | | | **MRSA (n = 17,090)** | | | |
| **Year** | **Δ** | **95% CI** | | **p** | **Δ** | **95% CI** | | **p** | **Δ** | **95% CI** | | **p** | **Δ** | **95% CI** | | **p** |
| 2020 | -30% | -39% | \| -20% | <0.001 | -35% | -41% | \| -29% | <0.001 | -40% | -47% | \| -32% | <0.001 | -33% | -41% | \| -25% | <0.001 |
| 2021 | -23% | -36% | \| -8% | 0.004 | -31% | -38% | \| -22% | <0.001 | -61% | -67% | \| -53% | <0.001 | -25% | -35% | \| -14% | <0.001 |
| 2022 | +32% | +6% | \| +64% | 0.013 | +6% | -9% | \| +23% | 0.453 | -48% | -58% | \| -36% | <0.001 | -20% | -32% | \| -6% | 0.008 |
| **Annual model with inpatient count** | | | | | | | | | | | | | | | | |
|  | **CRA (*n* = 4,079)** | | | | **CRKP (*n* = 9,347)** | | | | **CREC (*n* = 4,686)** | | | | **MRSA (n = 17,090)** | | | |
| **Year** | **Δ** | **95% CI** | | **p** | **Δ** | **95% CI** | | **p** | **Δ** | **95% CI** | | **p** | **Δ** | **95% CI** | | **p** |
| 2020 | -20% | -31% | \| -8% | 0.002 | -26% | -33% | \| -19% | <0.001 | -31% | -39% | \| -22% | <0.001 | -23% | -32% | \| -14% | <0.001 |
| 2021 | -12% | -27% | \| +6% | 0.168 | -20% | -29% | \| -10% | <0.001 | -55% | -62% | \| -46% | <0.001 | -14% | -25% | \| -1% | 0.036 |
| 2022 | +54% | +24% | \| +92% | <0.001 | +24% | +7% | \| +44% | 0.005 | -39% | -51% | \| -25% | <0.001 | +5% | -19% | \| +11% | 0.519 |
| **Only infected** | | | | | | | | | | | | | | | | |
| **Annual model without inpatient count** | | | | | | | | | | | | | | | | |
|  | **CRA (*n* = 1,951)** | | | | **CRKP (*n* = 4,179)** | | | | **CREC (*n* = 1833)** | | | | **MRSA (n = 17,090)** | | | |
| **Year** | **Δ** | **95% CI** | | **p** | **Δ** | **95% CI** | | **p** | **Δ** | **95% CI** | | **p** | **Δ** | **95% CI** | | **p** |
| 2020 | -43% | -54% | \| -29% | <0.001 | -40% | -48% | \| -31% | <0.001 | -47% | -56% | \| -35% | <0.001 | -33% | -41% | -25% | <0.001 |
| 2021 | -35% | -50% | \| -16% | 0.001 | -37% | -47% | \| -24% | <0.001 | -70% | -77% | \| -61% | <0.001 | -25% | -35% | -14% | <0.001 |
| 2022 | +21% | -12% | \| +65% | 0.241 | +1% | -20% | \| +26% | 0.966 | -64% | -75% | \| -50% | <0.001 | -20% | -32% | -6% | 0.008 |
| **Annual model with inpatient count** | | | | | | | | | | | | | | | | |
|  | **CRA (*n* = 1,951)** | | | | **CRKP (*n* = 4,179)** | | | | **CREC (*n* = 1833)** | | | | **MRSA (n = 17,090)** | | | |
| **Year** | **Δ** | **95% CI** | | **p** | **Δ** | **95% CI** | | **p** | **Δ** | **95% CI** | | **p** | **Δ** | **95% CI** | | **p** |
| 2020 | -34% | -47% | \| -19% | <0.001 | -31% | -40% | \| -21% | <0.001 | -39% | -50% | \| -26% | <0.001 | -23% | -32% | \| -14% | <0.001 |
| 2021 | -25% | -43% | \| -3% | 0.028 | -27% | -39% | \| -12% | <0.001 | -66% | -74% | \| -55% | <0.001 | -14% | -25% | \| -1% | 0.036 |
| 2022 | +41% | +3% | \| +93% | 0.031 | +18% | -6% | \| +47% | 0.152 | -58% | -70% | \| -42% | <0.001 | +5% | -19% | \| +11% | 0.519 |
| **Infected and colonised, weekly model with seasonality** | | | | | | | | | | | | | | | | |
| **Weekly model without inpatient count** | | | | | | | | | | | | | | | | |
|  | **CRA (*n* = 4,079)** | | | | **CRKP (*n* = 9,347)** | | | | **CREC (*n* = 4,686)** | | | | **MRSA (n = 17,090)** | | | |
| **Year** | **Δ** | **95% CI** | | **p** | **Δ** | **95% CI** | | **p** | **Δ** | **95% CI** | | **p** | **Δ** | **95% CI** | | **p** |
| 2020 | -31% | -40% | \| -20% | <0.001 | -34% | -40% | \| -28% | <0.001 | -37% | -44% | \| -29% | <0.001 | -34% | -39% | \| -29% | <0.001 |
| 2021 | -22% | -35% | \| -7% | 0.006 | -26% | -35% | \| -17% | <0.001 | -56% | -63% | \| -48% | <0.001 | -25% | -30% | \| -18% | <0.001 |
| 2022 | +35% | +9% | \| +67% | 0.006 | +15% | -1% | \| +33% | 0.065 | -40% | -51% | \| -27% | <0.001 | -19% | -26% | \| -11% | <0.001 |
| **Weekly model with inpatient count** | | | | | | | | | | | | | | | | |
|  | **CRA (*n* = 4,079)** | | | | **CRKP (*n* = 9,347)** | | | | **CREC (*n* = 4,686)** | | | | **MRSA (n = 17,090)** | | | |
| **Year** | **Δ** | **95% CI** | | **p** | **Δ** | **95% CI** | | **p** | **Δ** | **95% CI** | | **p** | **Δ** | **95% CI** | | **p** |
| 2020 | -20% | -31% | \| -8% | 0.001 | -24% | -31% | \| -17% | <0.001 | -28% | -36% | \| -18% | <0.001 | -24% | -29% | \| -18% | <0.001 |
| 2021 | -11% | -25% | \| +7% | 0.225 | -15% | -25% | \| -5% | 0.007 | -50% | -57% | \| -41% | <0.001 | -12% | -19% | \| -5% | 0.001 |
| 2022 | +58% | +27% | \| +96% | <0.001 | +34% | +16% | \| +55% | <0.001 | -30% | -43% | \| -14% | <0.001 | -3% | -12% | \| +6% | 0.45 |

Supplementary Table 3: Statutory surveillance data – Details of different statutory models, i.e. absolute numbers of observed notifications, hypothetically projected notifications and differences for statutory CRA, CRKP, CREC and MRSA notifications. Germany, 2017 – 2022.

| **Infected and colonised** | | | | | | | | | | | | |
| --- | --- | --- | --- | --- | --- | --- | --- | --- | --- | --- | --- | --- |
| **Annual model without inpatient count** | | | | | | | | | | | | |
|  | **CRA (*n* = 4,079)** | | | **CRKP (*n* = 9,347)** | | | **CREC (*n* = 4,686)** | | | **MRSA (*n* = 17,090)** | | |
| **Year** | **Observed** | **Projection** | **Difference** | **Observed** | **Projection** | **Difference** | **Observed** | **Projection** | **Difference** | **Observed** | **Projection** | **Difference** |
| 2015 | NA | NA | NA | NA | NA | NA | NA | NA | NA | 3,570 | 3,498 | NA |
| 2016 | NA | NA | NA | NA | NA | NA | NA | NA | NA | 3,197 | 3,043 | NA |
| 2017 | 786 | 797 | NA | 1,356 | 1,337 | NA | 568 | 561 | NA | 2,843 | 2,618 | NA |
| 2018 | 780 | 758 | NA | 1,441 | 1,478 | NA | 715 | 729 | NA | 2,446 | 2,216 | NA |
| 2019 | 711 | 722 | NA | 1,653 | 1,634 | NA | 955 | 948 | NA | 1,838 | 1,856 | NA |
| 2020 | 480 | 687 | -207 | 1,169 | 1,807 | -638 | 739 | 1,232 | -493 | 1,126 | 1,549 | -565 |
| 2021 | 501 | 654 | -153 | 1,389 | 1,997 | -608 | 631 | 1,602 | -971 | 1,078 | 1,290 | -366 |
| 2022 | 821 | 622 | 199 | 2,339 | 2,208 | 131 | 1,078 | 2,082 | -1004 | 992 | 1,073 | -242 |
| **Annual model with inpatient count** | | | | | | | | | | | | |
|  | **CRA (*n* = 4,079)** | | | **CRKP (*n* = 9,347)** | | | **CREC (*n* = 4,686)** | | | **MRSA (*n* = 17,090)** | | |
| **Year** | **Observed** | **Projection** | **Difference** | **Observed** | **Projection** | **Difference** | **Observed** | **Projection** | **Difference** | **Observed** | **Projection** | **Difference** |
| 2015 | NA | NA | NA | NA | NA | NA | NA | NA | NA | 3,570 | 3,480 | NA |
| 2016 | NA | NA | NA | NA | NA | NA | NA | NA | NA | 3,197 | 3,073 | NA |
| 2017 | 786 | 798 | NA | 1,356 | 1,340 | NA | 568 | 562 | NA | 2,843 | 2,629 | NA |
| 2018 | 780 | 756 | NA | 1,441 | 1,474 | NA | 715 | 727 | NA | 2,446 | 2,210 | NA |
| 2019 | 711 | 723 | NA | 1,653 | 1,637 | NA | 955 | 949 | NA | 1,838 | 1,858 | NA |
| 2020 | 480 | 600 | -120 | 1,169 | 1,577 | -408 | 739 | 1,075 | -336 | 1,126 | 1,350 | -343 |
| 2021 | 501 | 569 | -68 | 1,389 | 1,736 | -347 | 631 | 1,392 | -761 | 1,078 | 1,118 | -170 |
| 2022 | 821 | 532 | 289 | 2,339 | 1,884 | 455 | 1,078 | 1,775 | -697 | 992 | 912 | -52 |
| **Infected only** | | | | | | | | | | | | |
| **Annual model without inpatient count** | | | | | | | | | | | | |
|  | **CRA (*n* = 1,951)** | | | **CRKP (*n* = 4,179)** | | | **CREC (*n* = 1833)** | | | **MRSA (*n* = 17,090)** | | |
| **Year** | **Observed** | **Projection** | **Difference** | **Observed** | **Projection** | **Difference** | **Observed** | **Projection** | **Difference** | **Observed** | **Projection** | **Difference** |
| 2015 | NA | NA | NA | NA | NA | NA | NA | NA | NA | 3,570 | 3,498 | NA |
| 2016 | NA | NA | NA | NA | NA | NA | NA | NA | NA | 3,197 | 3,043 | NA |
| 2017 | 346 | 358 | NA | 591 | 591 | NA | 198 | 194 | 218 | 2,843 | 2,618 | NA |
| 2018 | 383 | 359 | NA | 664 | 665 | NA | 270 | 278 | 297 | 2,446 | 2,216 | NA |
| 2019 | 347 | 359 | NA | 748 | 748 | NA | 402 | 398 | 435 | 1,838 | 1,856 | NA |
| 2020 | 206 | 360 | -154 | 505 | 841 | -336 | 304 | 570 | 663 | 1,126 | 1,549 | -565 |
| 2021 | 234 | 360 | -126 | 601 | 946 | -345 | 243 | 817 | 1014 | 1,078 | 1,290 | -366 |
| 2022 | 435 | 361 | 74 | 1,070 | 1,065 | 5 | 416 | 1,169 | 1547 | 992 | 1,073 | -242 |
| **Annual model with inpatient count** | | | | | | | | | | | | |
|  | **CRA (*n* = 1,951)** | | | **CRKP (*n* = 4,179)** | | | **CREC (*n* = 1833)** | | | **MRSA (*n* = 17,090)** | | |
| **Year** | **Observed** | **Projection** | **Difference** | **Observed** | **Projection** | **Difference** | **Observed** | **Projection** | **Difference** | **Observed** | **Projection** | **Difference** |
| 2015 | NA | NA | NA | NA | NA | NA | NA | NA | NA | 3,570 | 3,480 | NA |
| 2016 | NA | NA | NA | NA | NA | NA | NA | NA | NA | 3,197 | 3,073 | NA |
| 2017 | 346 | 359 | NA | 591 | 592 | NA | 198 | 194 | NA | 2,843 | 2,629 | NA |
| 2018 | 383 | 358 | NA | 664 | 662 | NA | 270 | 277 | NA | 2,446 | 2,210 | NA |
| 2019 | 347 | 360 | NA | 748 | 749 | NA | 402 | 398 | NA | 1,838 | 1,858 | NA |
| 2020 | 206 | 314 | -108 | 505 | 734 | -229 | 304 | 497 | -193 | 1,126 | 1,350 | -343 |
| 2021 | 234 | 313 | -79 | 601 | 823 | -222 | 243 | 709 | -466 | 1,078 | 1,118 | -170 |
| 2022 | 435 | 308 | 127 | 1,070 | 909 | 161 | 416 | 996 | -580 | 992 | 912 | -52 |
| **Infected and colonised, weekly model with seasonality** | | | | | | | | | | | | |
| **Weekly model without inpatient count** | | | | | | | | | | | | |
|  | **CRA (*n* = 4,079)** | | | **CRKP (*n* = 9,347)** | | | **CREC (*n* = 4,686)** | | | **MRSA (*n* = 17,090)** | | |
| **Year** | **Observed** | **Projection** | **Difference** | **Observed** | **Projection** | **Difference** | **Observed** | **Projection** | **Difference** | **Observed** | **Projection** | **Difference** |
| 2015 | NA | NA | NA | NA | NA | NA | NA | NA | NA | 3,570 | 3,728 | NA |
| 2016 | NA | NA | NA | NA | NA | NA | NA | NA | NA | 3,197 | 3,178 | NA |
| 2017 | 786 | 801 | NA | 1,356 | 1,337 | NA | 568 | 561 | NA | 2,843 | 2,709 | NA |
| 2018 | 780 | 758 | NA | 1,441 | 1,478 | NA | 715 | 729 | NA | 2,446 | 2,310 | NA |
| 2019 | 711 | 718 | NA | 1,653 | 1,634 | NA | 955 | 948 | NA | 1,838 | 1,969 | NA |
| 2020 | 480 | 691 | -211 | 1,169 | 1,769 | -600 | 739 | 1,171 | -432 | 1,126 | 1,707 | -581 |
| 2021 | 501 | 643 | -142 | 1,389 | 1,883 | -494 | 631 | 1,441 | -810 | 1,078 | 1,427 | -349 |
| 2022 | 821 | 609 | 212 | 2,339 | 2,040 | 299 | 1,078 | 1,802 | -724 | 992 | 1,217 | -225 |
| **Weekly model with inpatient count** | | | | | | | | | | | | |
|  | **CRA (*n* = 4,079)** | | | **CRKP (*n* = 9,347)** | | | **CREC (*n* = 4,686)** | | | **MRSA (*n* = 17,090)** | | |
| **Year** | **Observed** | **Projection** | **Difference** | **Observed** | **Projection** | **Difference** | **Observed** | **Projection** | **Difference** | **Observed** | **Projection** | **Difference** |
| 2015 | NA | NA | NA | NA | NA | NA | NA | NA | NA | 3,570 | 3,704 | NA |
| 2016 | NA | NA | NA | NA | NA | NA | NA | NA | NA | 3,197 | 3,206 | NA |
| 2017 | 786 | 802 | NA | 1,356 | 1,340 | NA | 568 | 562 | NA | 2,843 | 2,718 | NA |
| 2018 | 780 | 756 | NA | 1,441 | 1,474 | NA | 715 | 727 | NA | 2,446 | 2,300 | NA |
| 2019 | 711 | 719 | NA | 1,653 | 1,637 | NA | 955 | 949 | NA | 1,838 | 1,965 | NA |
| 2020 | 480 | 603 | -123 | 1,169 | 1,544 | -375 | 739 | 1,021 | -282 | 1,126 | 1,481 | -355 |
| 2021 | 501 | 560 | -59 | 1,389 | 1,637 | -248 | 631 | 1,251 | -620 | 1,078 | 1,230 | -152 |
| 2022 | 821 | 520 | 301 | 2,339 | 1,740 | 599 | 1,078 | 1,535 | -457 | 992 | 1,027 | -35 |


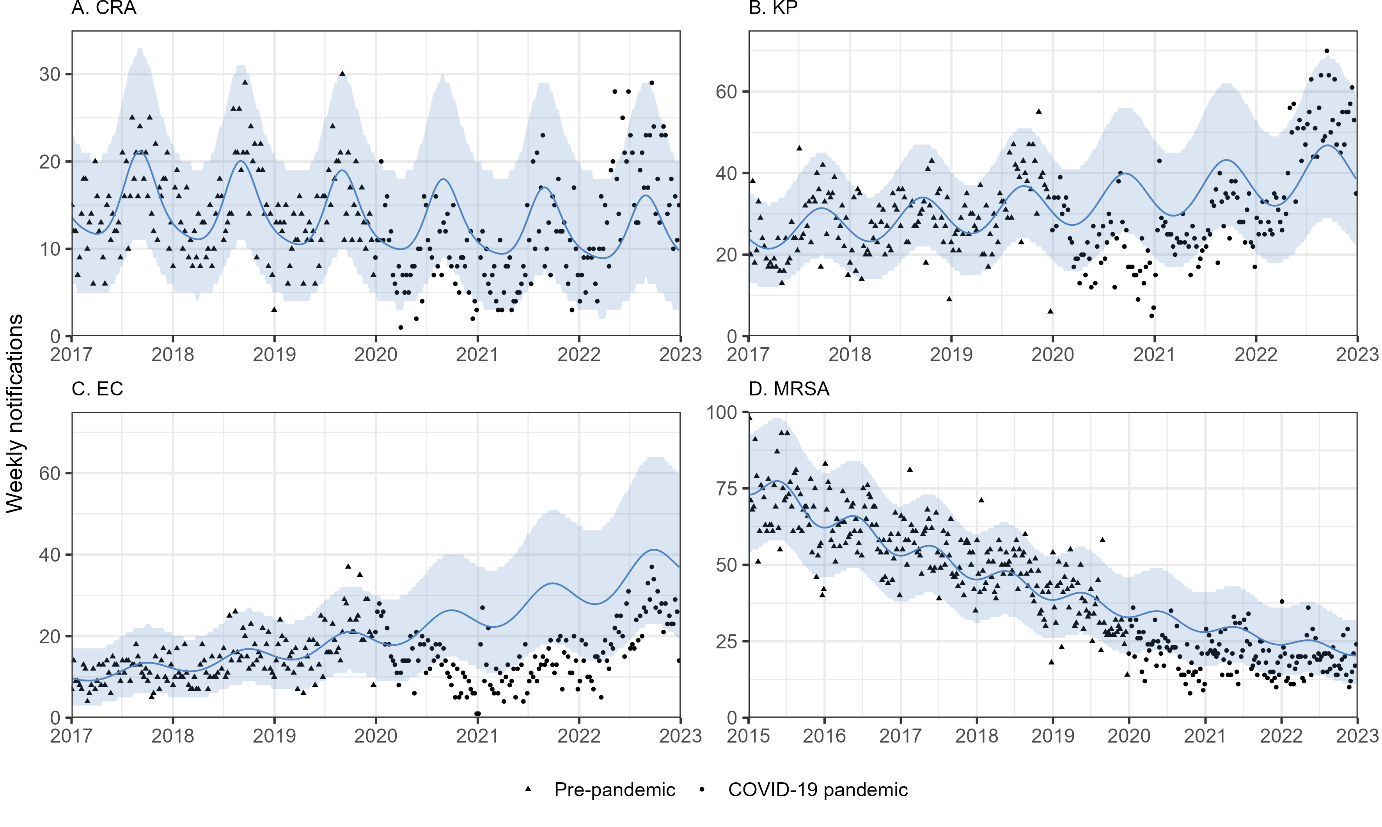

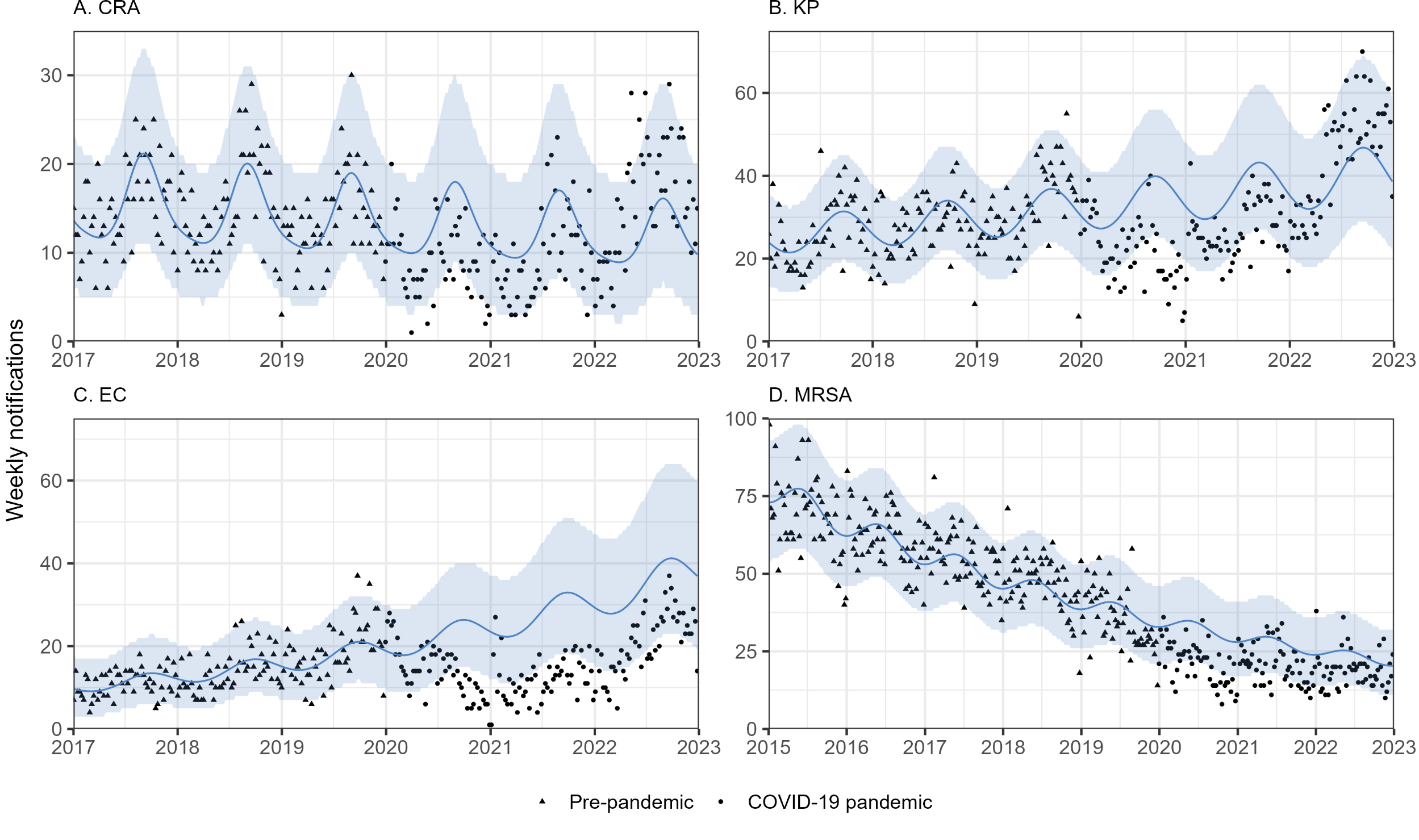

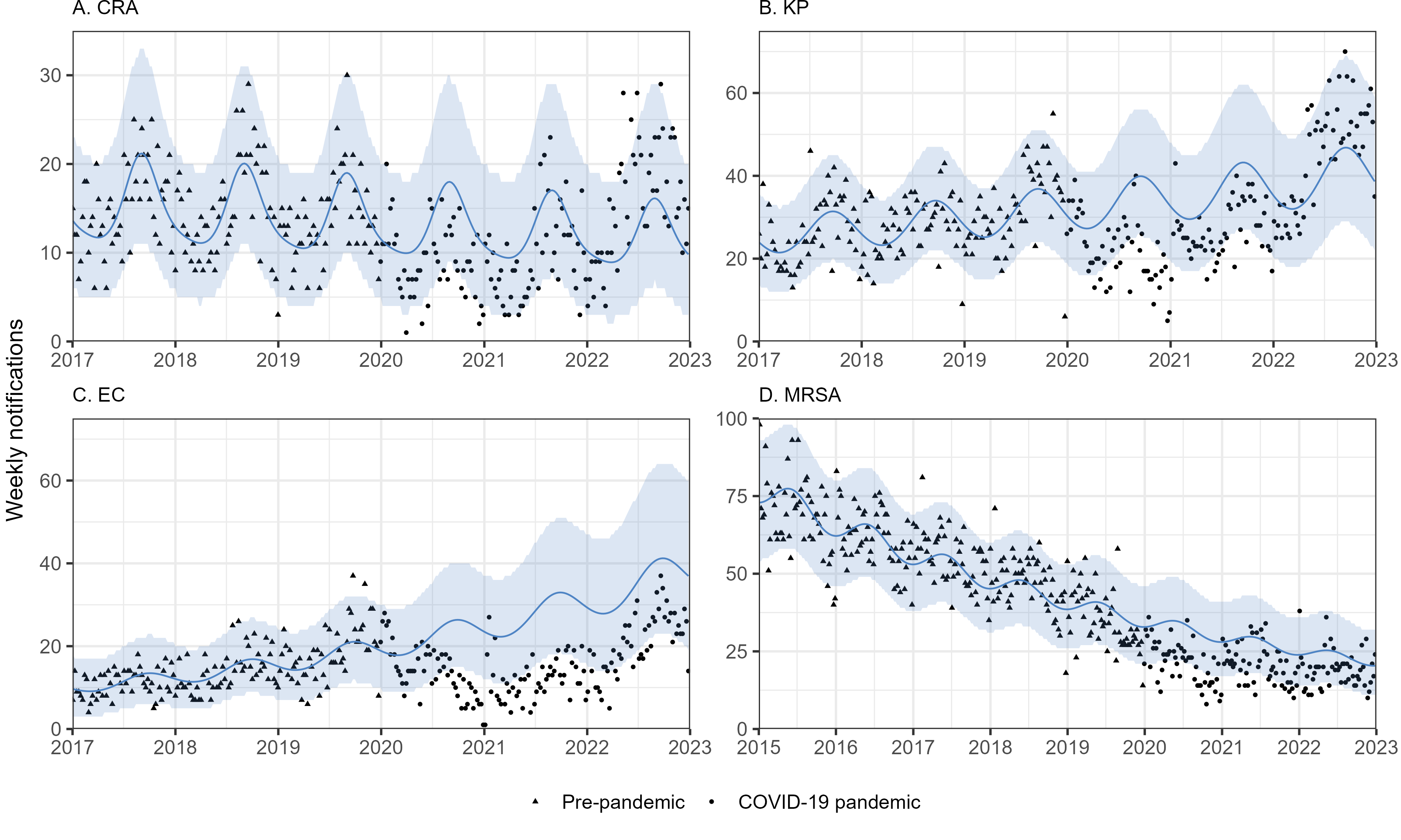

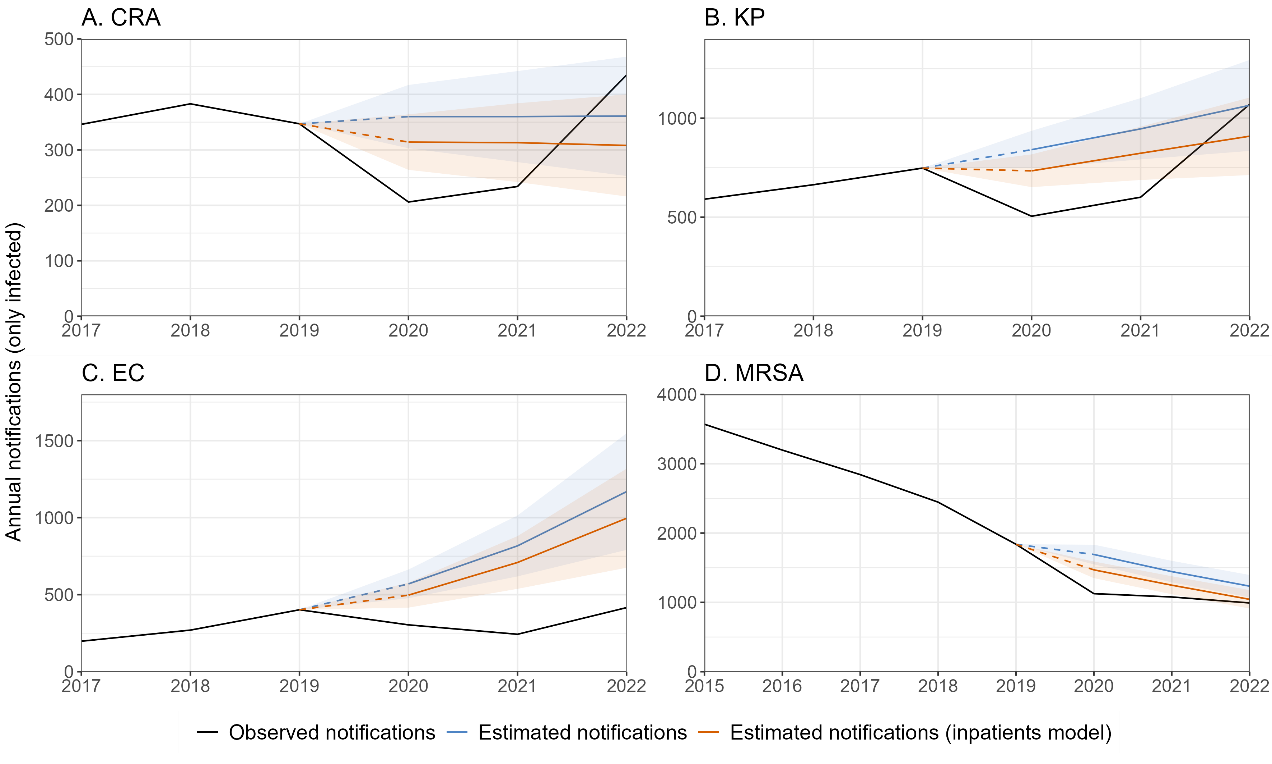

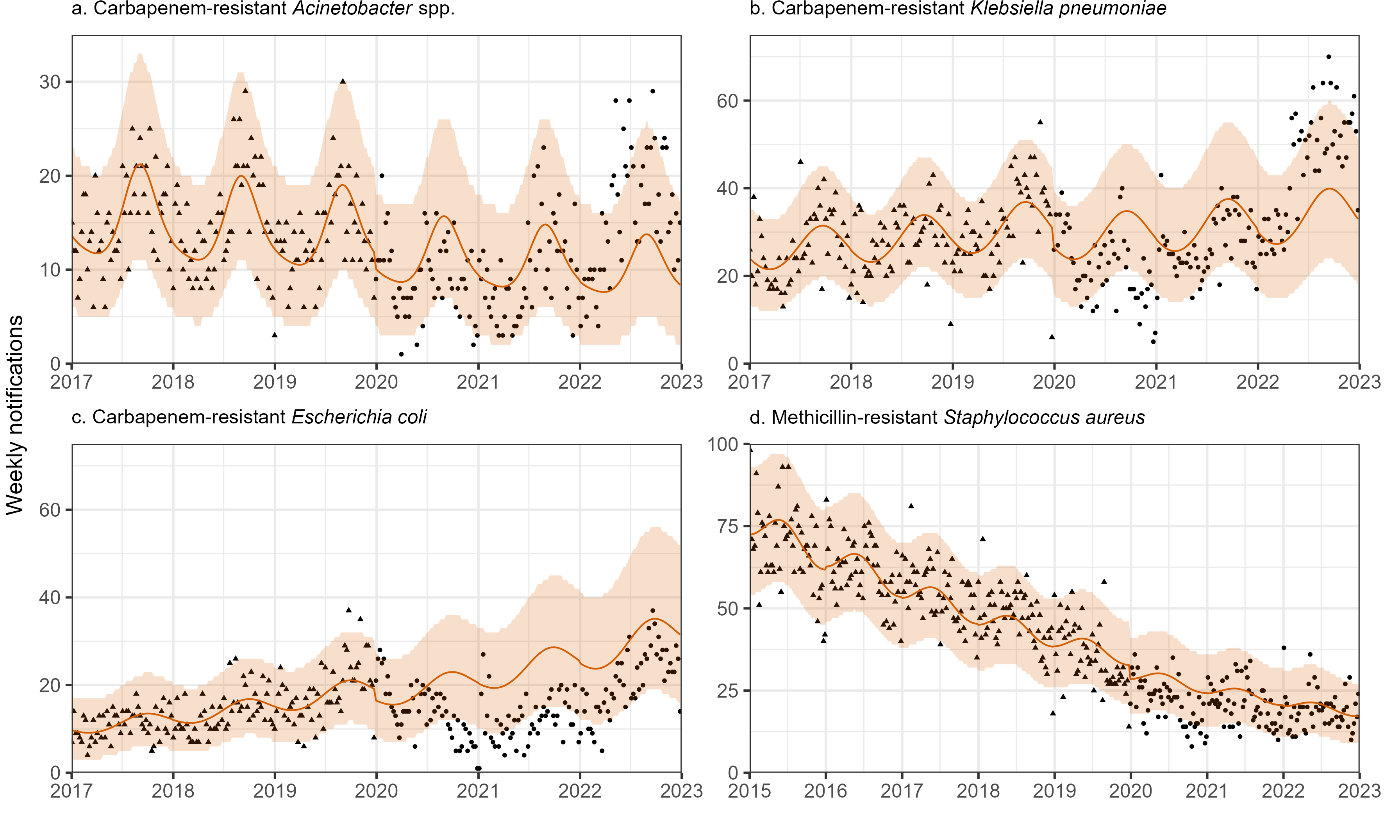

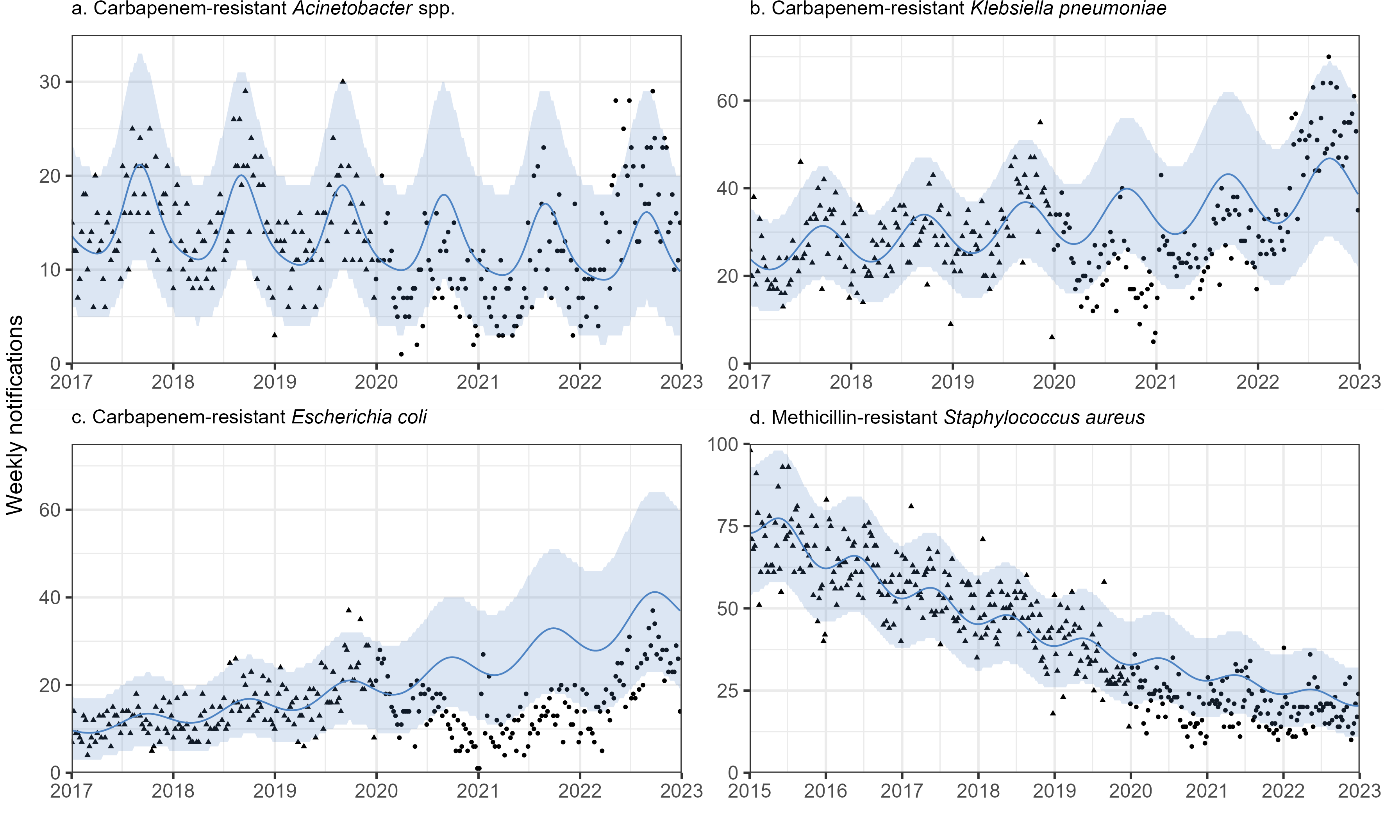


Supplementary Figure 10: Statutory surveillance data – Results of weekly models with seasonality, without or with inpatient offset, i.e. comparison of observed notification numbers and hypothetically projected notification numbers without the COVID-19 pandemic for statutory notifications of a. CRA (n = 4,079), b. CRKP (n = 9,347), c. CREC (n = 4,686) and d. MRSA (n = 17,090). Please note the different y-axis scales. Please note the different time frame for MRSA notifications. Germany, 2015 – 2022.

Supplementary Table 4: Antibiotic Resistance Surveillance (ARS) data – Results of annual models for resistant isolates of CRA, CRKP, CREC and MRSA as well as all isolates of Acinetobacter spp. (ACB), Klebsiella pneumonia (KP), Escherichia coli (EC) and Staphylococcus aureus (SA) in ARS. Incidence rate ratios were interpreted as relative changes (Δ) per pandemic year, i.e. as relative reductions (negative values) or increases (positive values) of observed annual isolate numbers relative to hypothetically projected isolate numbers without the COVID-19 pandemic. Statistical significance indicated as 95% confidence intervals (95% CI) and p-values (p). Germany, 2017 – 2022.

| **Resistant isolates** | | | | | | | | | | | | | | | | |
| --- | --- | --- | --- | --- | --- | --- | --- | --- | --- | --- | --- | --- | --- | --- | --- | --- |
| **Basic annual model** | | | | | | | | | | | | | | | | |
|  | **CRA (*n* = 3,218)** | | | | **CRKP (*n* = 3,258)** | | | | **CREC (*n* = 1,304)** | | | | **MRSA (*n* = 86,752)** | | | |
| **Year** | **Δ** | **95% CI** | | **p** | **Δ** | **95% CI** | | **p** | **Δ** | **95% CI** | | **p** | **Δ** | **95% CI** | | **p** |
| 2020 | -7% | -20% | \| 9% | 0.371 | -27% | -38% | \| -15% | <0.001 | +3% | -20% | \| 32% | 0.823 | -21% | -26% | \| -16% | <0.001 |
| 2021 | -12% | -29% | \| 8% | 0.209 | -16% | -32% | \| 3% | 0.097 | +6% | -25% | \| 48% | 0.756 | -26% | -32% | \| -19% | <0.001 |
| 2022 | +36% | +6% | \| 75% | 0.015 | +15% | -11% | \| 49% | 0.286 | -52% | -70% | \| -25% | 0.001 | -20% | -29% | \| -11% | <0.001 |
| **Resistant isolates (only infections based on sample material)** | | | | | | | | | | | | | | | | |
| **Basic annual model** | | | | | | | | | | | | | | | | |
|  | **CRA (*n* = 2,003)** | | | | **CRKP (*n* = 2,420)** | | | | **CREC (*n* = 1,091)** | | | | **MRSA (*n* = 53,108)** | | | |
| **Year** | **Δ** | **95% CI** | | **p** | **Δ** | **95% CI** | | **p** | **Δ** | **95% CI** | | **p** | **Δ** | **95% CI** | | **p** |
| 2020 | +1% | -17% | \| 24% | 0.893 | -27% | -39% | \| -11% | 0.001 | +11% | -16% | \| 46% | 0.484 | -21% | -26% | \| -16% | <0.001 |
| 2021 | +1% | -22% | \| 31% | 0.952 | -18% | -36% | \| 5% | 0.111 | +31% | -10% | \| 91% | 0.155 | -26% | -32% | \| -19% | <0.001 |
| 2022 | +58% | +14% | \| 117% | 0.005 | +7% | -21% | \| 45% | 0.654 | -48% | -68% | \| -14% | 0.011 | -20% | -29% | \| -11% | <0.001 |
| **Resistant isolates with inpatient care type** | | | | | | | | | | | | | | | | |
| **Basic annual model** | | | | | | | | | | | | | | | | |
|  | **CRA (*n* = 2,570)** | | | | **CRKP (*n* = 2,954)** | | | | **CREC (*n* = 979)** | | | | **MRSA (*n* = 59,848)** | | | |
| **Year** | **Δ** | **95% CI** | | **p** | **Δ** | **95% CI** | | **p** | **Δ** | **95% CI** | | **p** | **Δ** | **95% CI** | | **p** |
| 2020 | -2% | -18% | \| 17% | 0.837 | -29% | -40% | \| -16% | <0.001 | +14% | -15% | \| 53% | 0.37 | -21% | -27% | \| -16% | <0.001 |
| 2021 | -6% | -26% | \| 18% | 0.594 | -15% | -32% | \| 6% | 0.145 | +22% | -18% | \| 81% | 0.316 | -21% | -28% | \| -14% | <0.001 |
| 2022 | +64% | +24% | \| 117% | <0.001 | +20% | -8% | \| 58% | 0.183 | -41% | -65% | \| -1% | 0.045 | -14% | -23% | \| -3% | 0.012 |
| **Resistant isolates with outpatient care type** | | | | | | | | | | | | | | | | |
| **Basic annual model** | | | | | | | | | | | | | | | | |
|  | **CRA (*n* = 648)** | | | | **CRKP (*n* = 303)** | | | | **CREC (*n* = 325)** | | | | **MRSA *(n* = 26,903)** | | | |
| **Year** | **Δ** | **95% CI** | | **p** | **Δ** | **95% CI** | | **p** | **Δ** | **95% CI** | | **p** | **Δ** | **95% CI** | | **p** |
| 2020 | -27% | -48% | \| 2% | 0.065 | -19% | -51% | \| 36% | 0.432 | -24% | -53% | \| 24% | 0.266 | -22% | -26% | \| -18% | <0.001 |
| 2021 | -39% | -61% | \| -5% | 0.029 | -30% | -65% | \| 38% | 0.3 | -32% | -65% | \| 33% | 0.262 | -36% | -40% | \| -31% | <0.001 |
| 2022 | -40% | -66% | \| 5% | 0.075 | -30% | -71% | \| 67% | 0.421 | -74% | -89% | \| -37% | 0.003 | -34% | -40% | \| -29% | <0.001 |
| **All isolates (i.e. resistant and non-resistant)** | | | | | | | | | | | | | | | | |
| **Basic annual model** | | | | | | | | | | | | | | | | |
|  | ***ACB* (*n* = 90,598)** | | | | **KP (*n* = 516,917)** | | | | **EC (*n* = 2,552,201)** | | | | **SA (*n* = 1,030,515)** | | | |
| **Year** | **Δ** | **95% CI** | | **p** | **Δ** | **95% CI** | | **p** | **Δ** | **95% CI** | | **p** | **Δ** | **95% CI** | | **p** |
| 2020 | -14% | -16% | \| -11% | <0.001 | -10% | -11% | \| -9% | <0.001 | -7% | -7% | \| -6% | <0.001 | -13% | -14% | \| -12% | <0.001 |
| 2021 | -20% | -23% | \| -17% | <0.001 | -15% | -16% | \| -13% | <0.001 | -9% | -10% | \| -8% | <0.001 | -18% | -19% | \| -17% | <0.001 |
| 2022 | -25% | -28% | \| -21% | <0.001 | -21% | -22% | \| -19% | <0.001 | -13% | -15% | \| -12% | <0.001 | -22% | -23% | \| -20% | <0.001 |

Supplementary Table 5: Antibiotic Resistance Surveillance (ARS) data – Details of different ARS models, i.e. absolute numbers of observed isolates, hypothetically projected isolates and differences for resistant isolates of CRA, CRKP, CREC and MRSA as well as all isolates of Acinetobacter spp. (ACB), Klebsiella pneumonia (KP), Escherichia coli (EC) and Staphylococcus aureus (SA). Germany, 2017 – 2022.

| **Resistant isolates** | | | | | | | | | | |  |  |
| --- | --- | --- | --- | --- | --- | --- | --- | --- | --- | --- | --- | --- |
| **Standard model** | | | | | | | | | | | | |
|  | **CRA (n = 2,467)** | | | **CRKP (n = 2,230)** | | | **CREC (n = 1,149)** | | | **MRSA (n = 74,556)** | | |
| **Year** | **Observed** | **Projection** | **Difference** | **Observed** | **Projection** | **Difference** | **Observed** | **Projection** | **Difference** | **Observed** | **Projection** | **Difference** |
| 2017 | 680 | 657 | NA | 462 | 465 | NA | 133 | 131 | NA | 20,320 | 20,688 | NA |
| 2018 | 551 | 598 | NA | 509 | 503 | NA | 161 | 165 | NA | 19,185 | 18,515 | NA |
| 2019 | 568 | 545 | NA | 540 | 543 | NA | 208 | 206 | NA | 16,266 | 16,571 | NA |
| 2020 | 462 | 496 | -34 | 426 | 587 | -161 | 266 | 259 | 7 | 11,673 | 14,831 | -3,158 |
| 2021 | 396 | 452 | -56 | 532 | 634 | -102 | 342 | 324 | 18 | 9,854 | 13,274 | -3,420 |
| 2022 | 561 | 411 | 150 | 789 | 685 | 104 | 194 | 406 | -212 | 9,454 | 11,880 | -2,426 |
| **Resistant isolates (only infections based on sample material)** | | | | | | | | | | | | |
| **Standard model** | | | | | | | | | | | | |
|  | **CRA (*n* = 2,003)** | | | **CRKP (*n* = 2,420)** | | | **CREC (*n* = 1,091)** | | | **MRSA (*n* = 53,108)** | | |
| **Year** | **Observed** | **Projection** | **Difference** | **Observed** | **Projection** | **Difference** | **Observed** | **Projection** | **Difference** | **Observed** | **Projection** | **Difference** |
| 2017 | 409 | 406 | NA | 317 | 321 | NA | 108 | 111 | NA | 12,493 | 12,712 | NA |
| 2018 | 356 | 362 | NA | 366 | 359 | NA | 140 | 134 | NA | 11,616 | 11,218 | NA |
| 2019 | 326 | 323 | NA | 397 | 401 | NA | 160 | 163 | NA | 9,718 | 9,900 | NA |
| 2020 | 292 | 288 | 4 | 329 | 448 | -119 | 218 | 197 | 21 | 7,227 | 8,737 | -1,510 |
| 2021 | 259 | 257 | 2 | 411 | 501 | -90 | 314 | 239 | 75 | 6,142 | 7,710 | -1,568 |
| 2022 | 361 | 229 | 132 | 600 | 560 | 40 | 151 | 290 | -139 | 5,912 | 6,804 | -892 |
| **Resistant isolates with inpatient care type** | | | | | | | | | | | | |
| **Basic annual model** | | | | | | | | | | | | |
|  | **CRA (*n* = 2,570)** | | | **CRKP (*n* = 2,954)** | | | **CREC (*n* = 979)** | | | **MRSA (*n* = 59,848)** | | |
| **Year** | **Observed** | **Projection** | **Difference** | **Observed** | **Projection** | **Difference** | **Observed** | **Projection** | **Difference** | **Observed** | **Projection** | **Difference** |
| 2017 | 591 | 564 | NA | 428 | 429 | NA | 105 | 103 | NA | 14,601 | 14,864 | NA |
| 2018 | 433 | 487 | NA | 461 | 459 | NA | 120 | 123 | NA | 13,404 | 12,933 | NA |
| 2019 | 448 | 421 | NA | 490 | 491 | NA | 149 | 147 | NA | 11,040 | 11,252 | NA |
| 2020 | 357 | 364 | -7 | 374 | 525 | -151 | 201 | 176 | 25 | 7,695 | 9,789 | -2,094 |
| 2021 | 295 | 314 | -19 | 478 | 562 | -84 | 257 | 210 | 47 | 6,717 | 8,517 | -1,800 |
| 2022 | 446 | 271 | 175 | 723 | 601 | 122 | 147 | 251 | -104 | 6,391 | 7,410 | -1,019 |
| **Resistant isolates with outpatient care type** | | | | | | | | | | | | |
| **Basic annual model** | | | | | | | | | | | | |
|  | **CRA (*n* = 648)** | | | **CRKP (*n* = 303)** | | | **CREC (*n* = 325)** | | | **MRSA *(n* = 26,903)** | | |
| **Year** | **Observed** | **Projection** | **Difference** | **Observed** | **Projection** | **Difference** | **Observed** | **Projection** | **Difference** | **Observed** | **Projection** | **Difference** |
| 2017 | 89 | 94 | NA | 33 | 35 | NA | 28 | 28 | NA | 5,719 | 5,823 | NA |
| 2018 | 118 | 108 | NA | 48 | 43 | NA | 41 | 41 | NA | 5,780 | 5,571 | NA |
| 2019 | 120 | 125 | NA | 50 | 52 | NA | 59 | 59 | NA | 5,226 | 5,330 | NA |
| 2020 | 105 | 144 | -39 | 52 | 64 | -12 | 65 | 86 | -21 | 3,978 | 5,100 | -1,122 |
| 2021 | 101 | 166 | -65 | 54 | 78 | -24 | 85 | 124 | -39 | 3,137 | 4,879 | -1,742 |
| 2022 | 115 | 192 | -77 | 66 | 94 | -28 | 47 | 180 | -133 | 3,063 | 4,668 | -1,605 |
| **All isolates (i.e. resistant and non-resistant)** | | | | | | | | | | | | |
| **Standard model** | | | | | | | | | | | | |
|  | ***ACB* (*n* = 90,598)** | | | **KP (*n* = 516,917)** | | | **EC (*n* = 2,552,201)** | | | **SA (*n* = 1,030,515)** | | |
| **Year** | **Observed** | **Projection** | **Difference** | **Observed** | **Projection** | **Difference** | **Observed** | **Projection** | **Difference** | **Observed** | **Projection** | **Difference** |
| 2017 | 16,005 | 15,776 | NA | 77,904 | 78,021 | NA | 408,785 | 409,644 | NA | 182,239 | 182,819 | NA |
| 2018 | 15,724 | 16,181 | NA | 84,085 | 83,851 | NA | 426,129 | 424,384 | NA | 186,275 | 185,107 | NA |
| 2019 | 16,825 | 16,596 | NA | 89,999 | 90,116 | NA | 438,767 | 439,654 | NA | 186,836 | 187,423 | NA |
| 2020 | 14,658 | 17,022 | -2,364 | 87,314 | 96,850 | -9,536 | 424,673 | 455,473 | -30,800 | 165,531 | 189,769 | -24,238 |
| 2021 | 13,921 | 17,459 | -3,538 | 88,940 | 104,086 | -15,146 | 430,735 | 471,861 | -41,126 | 157,310 | 192,143 | -34,833 |
| 2022 | 13,465 | 17,907 | -4,442 | 88,675 | 111,863 | -23,188 | 423,112 | 488,839 | -65,727 | 152,324 | 194,548 | -42,224 |


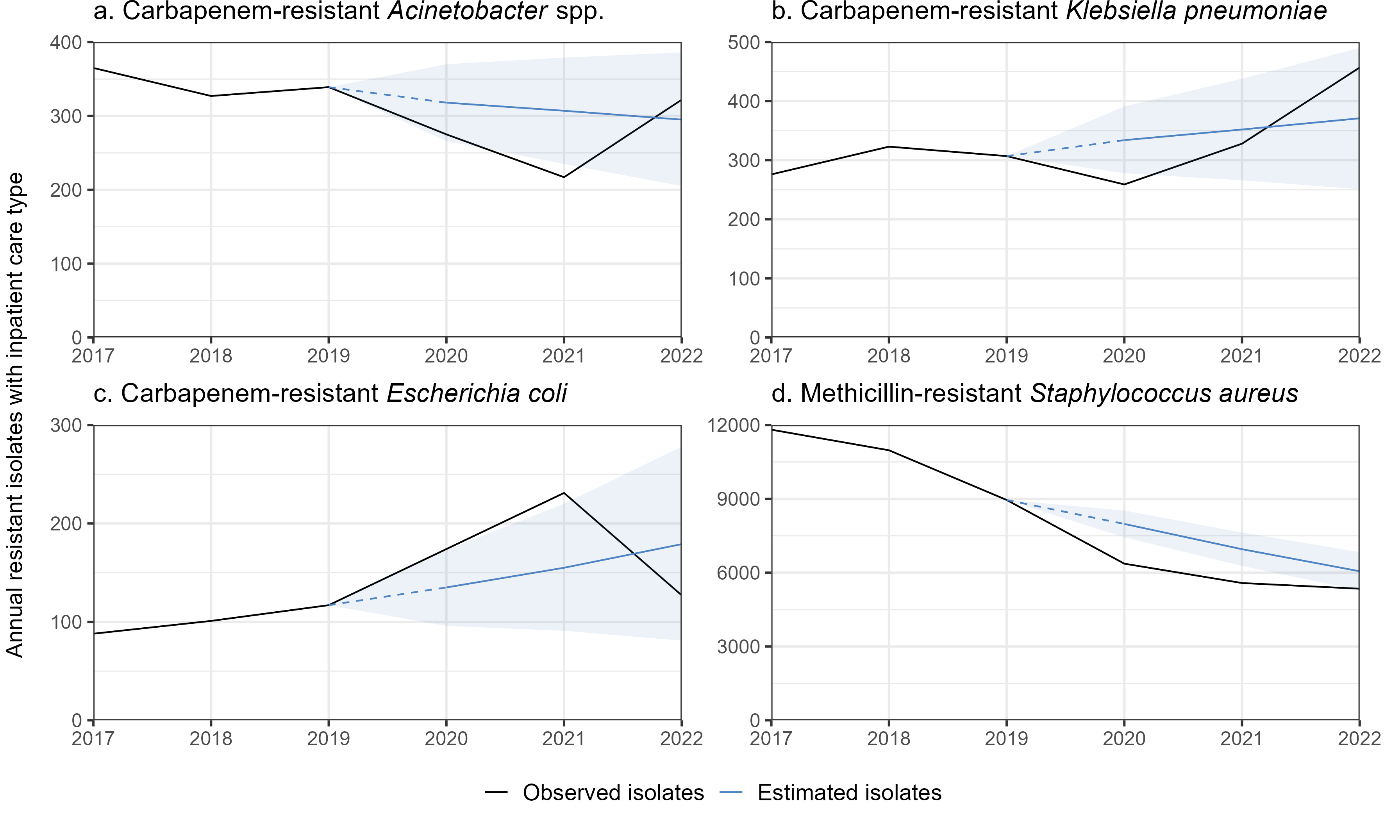


Supplementary Figure 11: Antibiotic Resistance Surveillance (ARS) data – Results of annual models for all resistant isolates with inpatient care type in ARS, i.e. comparison of observed isolates and hypothetically projected isolate numbers without the COVID-19 pandemic for a. CRA. (n = 2,570), b. CRKP (n = 2,954), c. CREC (n = 979) and d. MRSA (n = 59,848). Please note the different y-axis scales. Germany, 2017 – 2022.


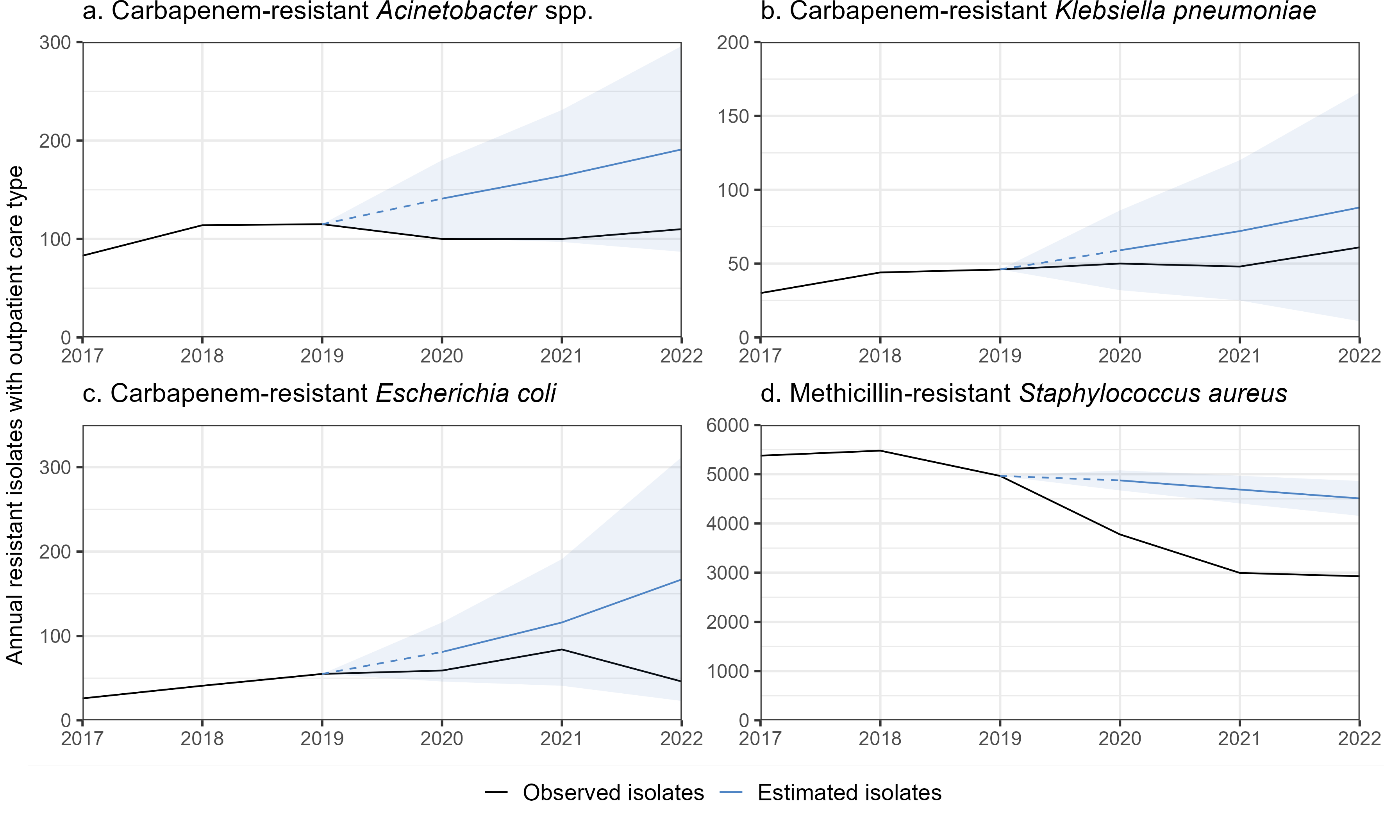


Supplementary Figure 12: Antibiotic Resistance Surveillance (ARS) data – Results of annual models for resistant isolates with outpatient care type in ARS, i.e. comparison of observed isolates and hypothetically projected isolate numbers without the COVID-19 pandemic for a. CRA. (n = 648), b. CRKP (n = 303), c. CREC (n = 325) and d. MRSA (n = 26,903). Please note the different y-axis scales. Germany, 2017 – 2022.


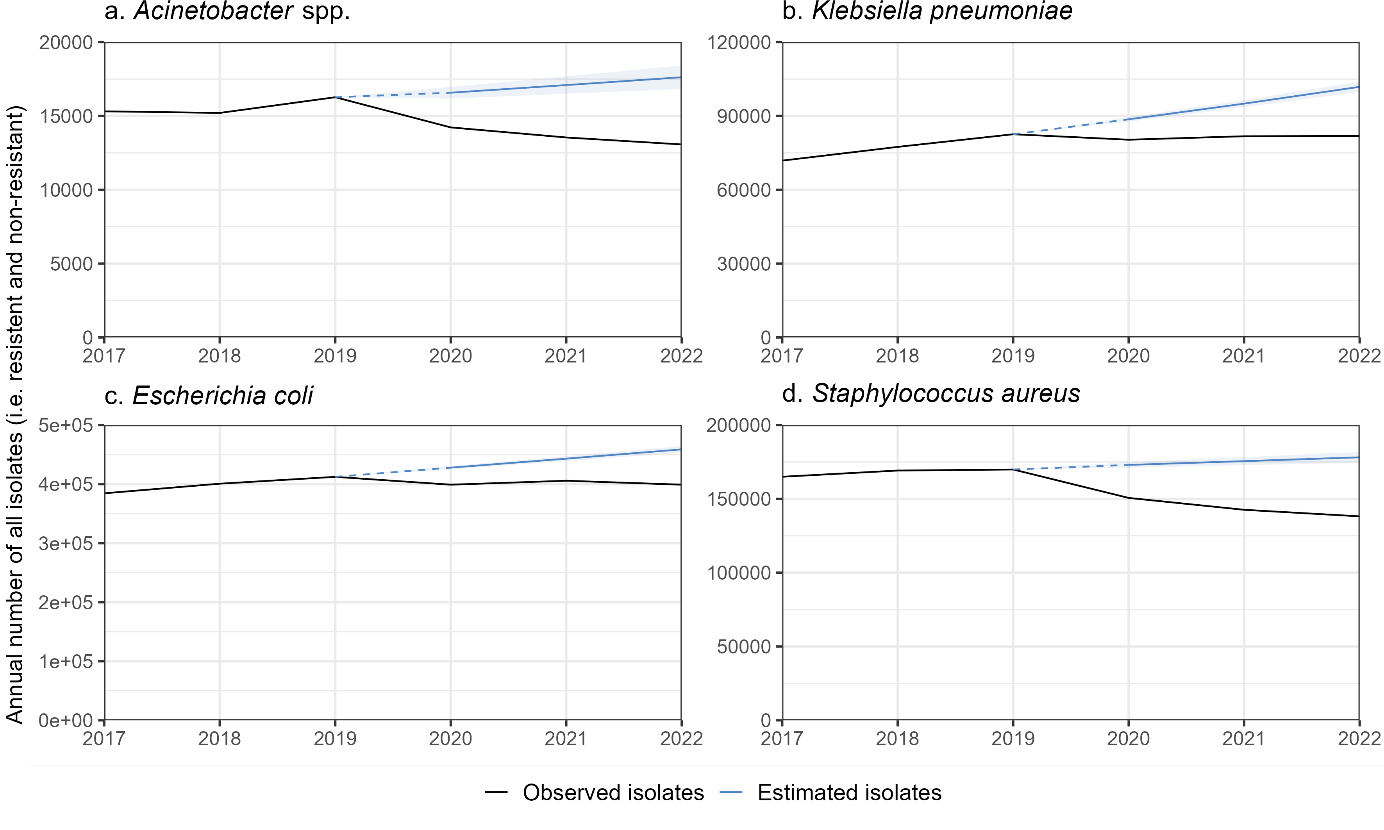


Supplementary Figure 13: Antibiotic Resistance Surveillance (ARS) data – Results of annual models for all isolates in ARS, i.e. comparison of observed isolate numbers and hypothetically projected isolate numbers without the COVID-19 pandemic for a. Acinetobacter spp. (n = 90,598), b. Klebsiella pneumoniae (n = 516,917), c. Escherichia coli (n = 2,552,201) and d. Staphylococcus aureus (n = 1,030,515). Please note the different y-axis scales. Germany, 2017 – 2022.


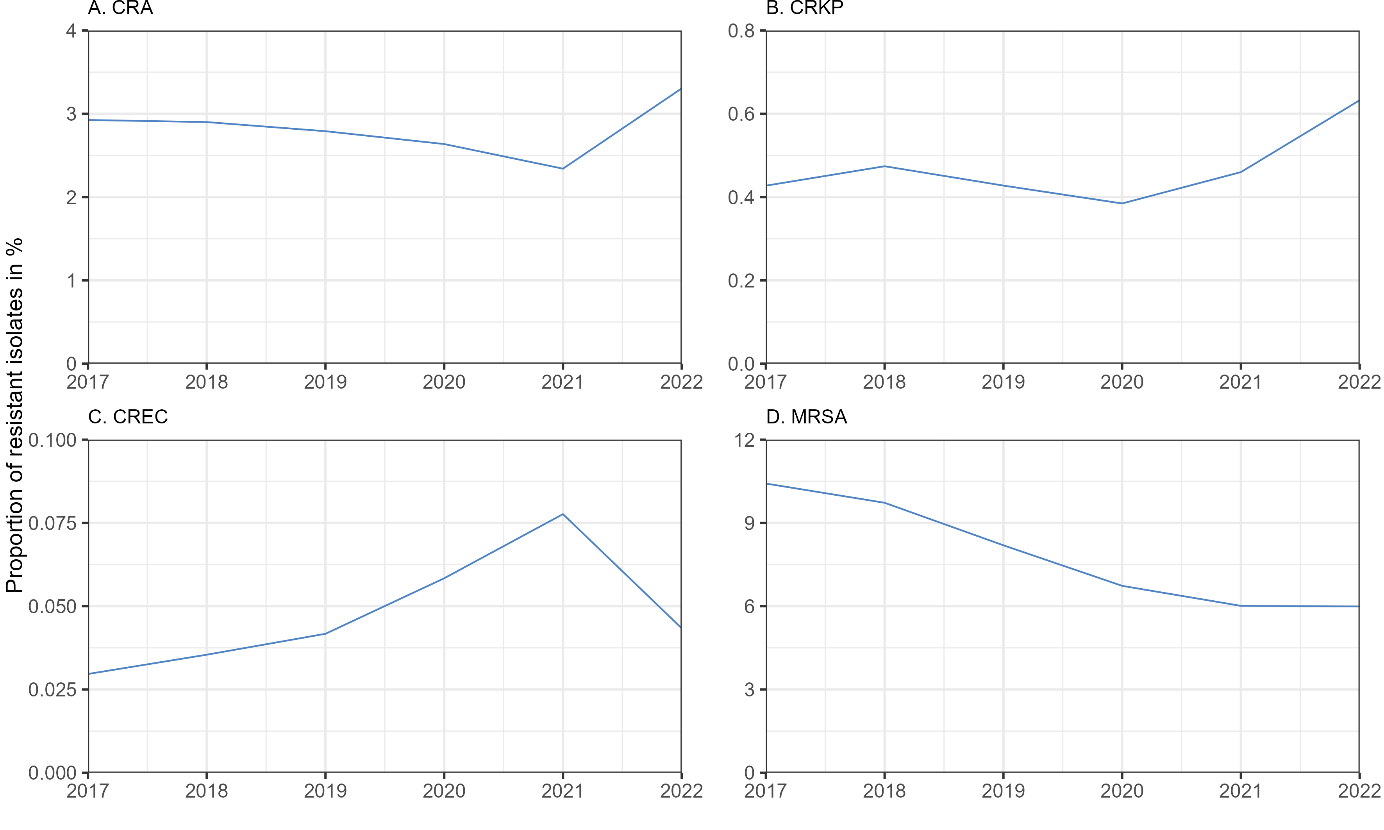

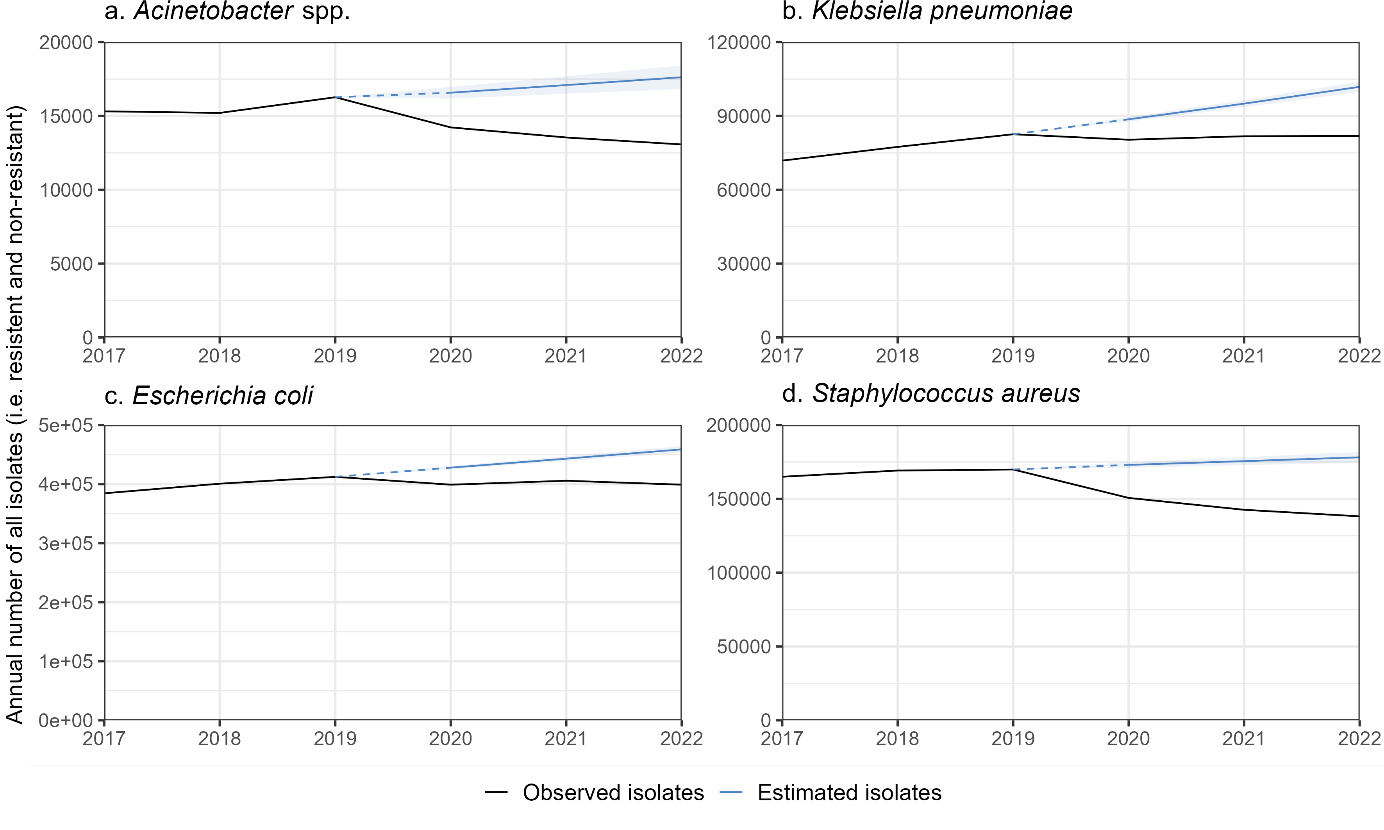

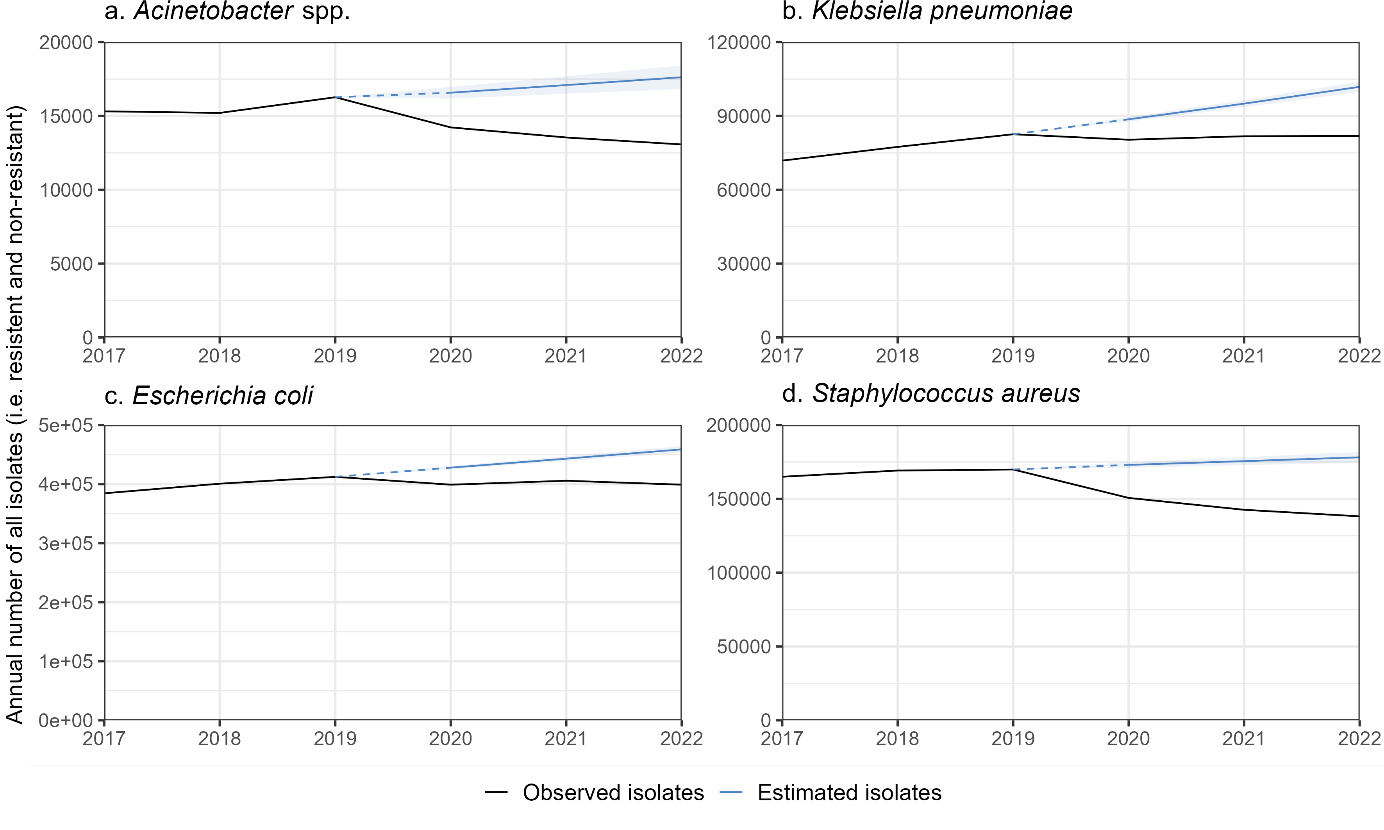


Supplementary Figure 14: Antibiotic Resistance Surveillance (ARS) data – Percentage of resistant isolates in ARS among all isolates for a. CRA (n = 2,467 / 90,598), b. CRKP (n = 2,230 / 516,917), c. CREC (n = 1,149 / 2,552,201) and d. MRSA (n = 74,556 / 1,030,515). Please note the different y-axis scales. Germany, 2017 – 2022.
